# Cerumenogram: a new frontier in cancer diagnosis in humans

**DOI:** 10.1038/s41598-019-48121-4

**Published:** 2019-08-13

**Authors:** João Marcos Gonçalves Barbosa, Naiara Zedes Pereira, Lurian Caetano David, Camilla Gabriela de Oliveira, Marina Ferraz Gontijo Soares, Melissa Ameloti Gomes Avelino, Anselmo Elcana de Oliveira, Engy Shokry, Nelson Roberto Antoniosi Filho

**Affiliations:** 1 Laboratory of Extraction and Separation Methods (LAMES), Institute of Chemistry, Federal University of Goiás (UFG), Campus II - Samambaia, 74690-900 Goiânia, GO Brazil; 2 Clinical Hospital, Federal University of Goiás (UFG), Campus I – Colemar Natal e Silva, 74605-020 Goiânia, GO Brazil; 3 Laboratory of Theoretical and Computational Chemistry (LQTC), Institute of Chemistry, Federal University of Goiás (UFG), Campus II - Samambaia, 74690-970 Goiânia, GO Brazil; 4 Present Address: Laboratory of Extraction and Separation Methods (LAMES), Institute of Chemistry, Federal University of Goiás (UFG), Campus II - Samambaia, 74690-900 Goiânia, GO Brazil

**Keywords:** Cancer, Cancer, Medicinal chemistry

## Abstract

Cancer is the deadliest human disease and the development of new diagnosis methods is important to increase the chances of a cure. In this work it was developed a new method, named here for the first time as cerumenogram, using cerumen (earwax) as a new biomatrix for diagnosis. Earwax samples collected from cancer patients (cancer group) and cancer-free patients (control group) were analyzed by Headspace/Gas Chromatography-Mass Spectrometry (HS/GC-MS), following with multivariate analysis steps to process the raw data generated. In total, 158 volatile organic metabolites (VOMs) were identified in the cerumen samples. The 27 selected as potential VOMs biomarkers for cancer provided 100% discrimination between the cancer and control groups. This new test can thus be routinely employed for cancer diagnoses that is non-invasive, fast, cheap, and highly accurate.

## Introduction

Cancer is a disease characterized by the uncontrollable rise of abnormal cells in the body that extend through organs and tissues and can spread all over an organism. One in three sudden deaths from non-communicable diseases is due to cancer^1^. The International Agency for Research on Cancer (IARC) estimated that there will be 18.1 million new cancer cases and 9.6 million cancer deaths in 2018 across 20 world regions^2^. Cancer incidences in humans are attributed to many factors, such as age, lifestyle behavior, hormones, and the exposure to environmental carcinogens^3,4^. Due to the many factors responsible for cancer diseases, and its huge ratio of incidence and mortality, early cancer diagnoses are urgently needed.

In a clinical routine, many conventional diagnostics tests depend on the suspected type of cancer in the patient, which include cytology, biopsy, blood tests, and physical exam. These tests are limited and often use painful and invasive methods; moreover, some types of cancer are not detected due the limitations of the tests. The existing non-invasive methods such as computed tomography (CT) and magnetic resonance imaging (MRI), are associated with high false-positive rates and harmful for patients, due to the risk of radiation exposure^5^.

Alternative approaches to these traditional diagnostics tools have been developed involving screening biomarkers in biological samples (biomatrices) using analytical methods. However, the search for biomarkers to develop a clinical diagnosis in early stages are widely associated with an invasive biomatrix, such as serum tests^6–8^ and clinical tumor samples^9–11^.

Many researchers in the bioanalytical field have focused attention on the development of new non-invasive techniques for cancer diagnoses^12^, which has led to the study of volatile organic metabolites (VOMs) present in human and animals biomatrices, as biomarkers for a wide range of diseases^13,14^. VOMs are a part of the metabolomics field that can also reflect the status of the body, and serve as a potential alternative to diagnose several diseases, because they can provide tests that are non-invasive, painless, cheap, fast, and easy^15^. VOMs are thermostable molecules that extend to a vast class of chemical compounds with a boiling point that goes from <0 °C (*e*.*g*. methane) to about 250 °C (*e*.*g*. limonene)^14,16^. VOMs are produced mainly for specific routes of a biochemical pathway (endogenous VOMs), or by exposure to an exogenous source (exogenous VOMs).

Endogenous VOMs are the aim in the studies to develop new diagnoses, because they are produced by reactive oxygen species (ROS) and free radicals excreted from the mitochondria in an oxidative stress process. ROS and liberated free radical attack many cellular structures (including RNA and DNA), and when accumulated in tissues, ROS destroys many different molecules, generating a wide range of VOMs classes^14^. ROS production is associated with the most inflammatory condition in cells, such as cancer diseases; therefore, ROS products are manifested at different levels in abnormal cell conditions and are accumulated in several biomatrices, such as urine, feces, sweat, tears, saliva, breath, and cerumen^15,17,18^.

Moreover, in the early stages of cancer, cancerous cells proliferate rapidly; consequently, adenosine monophosphate (AMP)-activated protein kinase is activated to accelerate the breakdown of lipids, for energy production to meet the energy requirement of cell proliferation and thus change the VOMs composition in body fluids^19^. Several studies have been conducted in recent years exploring the use of VOMs as biomarkers to identify cancer.

For this purpose, some diagnostic exams for cancer using blood, saliva, breath, fecal extract, and urine have been tested^20–24^, with some disadvantages such as affected by nutritional factors; low quantity and liability to diurnal variations of the VOMs composition; discriminations for polar or nonpolar VOMs depending on the polarity of the biomatrix; require time consuming pre-concentration steps; and effect of pH on composition^25^.

Nevertheless, despite the advantages of cerumen in relation to other biomatrices, such as no need for sample preparation, easy collect (painless), and less liability to external contamination (due to the location inside the auditory canal)^25^, it has been a neglected body secretion. Although cerumen is a concentrated form of excretion of polar and nonpolar metabolites, suitable for tracking long-term changes^26^. Only recently, has cerumen been used as a source of information about race, ethnicity and gender for individuals from East Asian, African, and Caucasian descent^27,28^. Recently, our research group successfully used cerumen VOMs to diagnose diabetes mellitus (DM) and differentiate between DM type I and II^29^. We have also investigated the viability of cerumen to diagnose intoxications and metabolic alterations in animals^25,30^, as well as for forensic applications as a monitoring biomatrix for drug abuse analysis^31^.

However, to date, cerumen has never been tested with the purpose of identifying and selecting potential volatile biomarkers for cancer. Thus, our work purposes the use of cerumen as a new biomatrix to diagnosis cancer in a new method named here, for the first time, as cerumenogram, with the principal objective to increase the accessibility to an accurate cancer diagnosis test, improving the chances of a cure. This identification is extremely important to advance the frontier for the development of new clinical diagnosis methods, allowing identification of cancer in a fast and conclusive way, using a non-invasive and easily collected biomatrix rich in both polar and nonpolar components. Thus, using Headspace/Gas Chromatography-Mass Spectrometry (HS/GC-MS) - a common technique found in many analytical laboratories around the world - analysis of cerumen volatile compounds, cerumenogram emerges as a new approach for cancer diagnosis.

## Results

We collected samples from healthy subjects (control group) and a cancer group within different ranges of first cancer diagnostic. The cancer patients were either in early (0–6 months), medium (6–12 months), and later (between 1 and 5 years or more) intervals of diagnostic. The cancer patients had been diagnosed with either Lymphoma (n = 11), Carcinoma (n = 28) or Leukemia (n = 13). Cancer samples were collected from individuals that have been in cancer treatment (chemotherapy, radiotherapy or both), and from patients that have not received any type of cancer treatment. Information about the date of samples’ collection, order of analysis and type of cancer of each sample is provided in the Supplementary Table 1 (Table S1). In addition, all information collected about the subjects from all groups in this study is summarized in Supplementary Table 2 (Table S2).

### Cerumen VOMs profile

The 102 cerumen samples collected from the cancer (n = 52) and control group (n = 50, healthy subjects, cancer free), were analyzed by HS/GC-MS. The 158 VOMs identified including organooxygen, carboxylic acids, organosulfur, hydrocarbons, and organonitrogen compounds. These compounds are summarized in Table S3 enumerate by the sequence of elution, absolute, and relative to the Internal Standard (IS) retention time. The fingerprint signals, of cerumen metabolomic profile, from GC-MS Total Ion Chromatograms (TIC) of the cancer group (divided into Carcinoma, Lymphoma, and Leukemia) contrasted with the control group are shown in Supplementary Fig. 1 (Fig. S1), where a wide range of compounds were identified, and the different TIC profile for each group is notable. Among the 158 VOMs, the largest group was the ketones with 36 compounds; followed by 24 hydrocarbons; 17 amines and amides derivatives; 14 esters and/or ethers; 14 aldehydes; 14 carboxylic acid; 13 furanic, lactones, and derivatives; 13 alcohols and derivatives; 8 pyran; 3 organosulfur; and 2 epoxides/oxabicyclo compounds.

### Statistical analysis

Using the data matrix of the 158 VOMs identified, we run a Hierarchical Cluster Analysis (HCA) labelling ethnicity/race as parameters and, as shown in Supplementary Fig. 2 (Fig. S2), there is no ethnicity/race effect on cerumen’s VOMs expression. Since the ethnicity/racial of the individuals has low influence in the types of the VOMs produced in cerumen, but influencing only in the VOMs concentration^27,28^, it was applied the binary model (VOMs absence/presence) aiming to avoid the effect of this factor in cancer detection. Besides that, the influence of gender on VOMs produced in cerumen was verified using gender’s parameters in the HCA. As shown in Supplementary Figs 3 and 4 (Figs S3 and S4), also cerumen samples from males and females are not discriminated by its VOMs profile. Thus, a binary model was constructed aiming to visualize and explore similarities and dissimilarities in the data. In this model, we utilized a Genetic Algorithm for a Partial Least Squares regression (GA-PLS) to select the most informative VOMs. This method is commonly used in very large data matrices and produces good improvement of the data^32^. In this case, the GA-PLS selected 27 out of 158 VOMs identified in cerumen analysis as a fingerprint for cancer diagnoses: 3 ketones; 3 ester and/or ether compounds; 1 aldehyde; 4 pyran compounds; 2 furanic, lactones, and derivatives; 1 epoxides/oxabicyclo; 3 hydrocarbons; 3 carboxylic acid; 3 alcohols and derivatives; 3 amines and amides derivatives; and 1 organosulfur compound.

**Figure 1:**
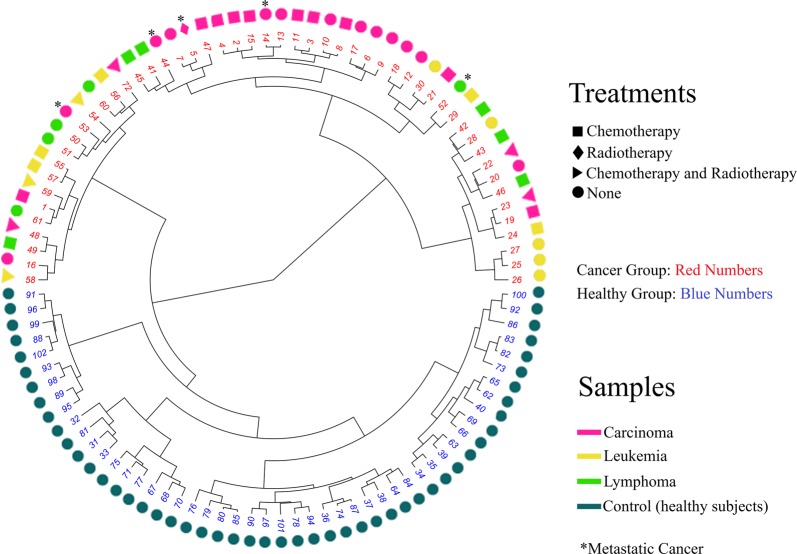
Circular dendrogram of the 102 cerumen samples using the 27 VOMs peak signals selected by GA-PLS from the 158 VOMs analyzed by HS/GC-MS. The circular dendrogram illustrates successful separation between the samples from the control (blue number) and cancer groups (red number). Samples from patients with different types of cancer are represented by different colors, and the oncological treatments are represented by different geometric symbols. Numbers on the branches in the HCA correspond to the order of samples analysis.

Using these 27 selected VOMs, we run a HCA and observed that all samples were correctly discriminated between cancer and control group, as shown in circular dendrogram in Fig. 1. However, there was no separation between the cancer types analyzed (Carcinoma, Lymphoma, and Leukemia), or between the type of treatment that the patients were previous submitted. Moreover, to verify the effect of the gender in the cancer samples discrimination, we run a HCA using these 27 potential biomarkers to observe any type of over-classification by gender on cancer identification. As shown in the Supplementary Figs 5 and 6 (Figs S5 and S6), there is no gender discrimination in the samples by these 27 cerumen VOMs profile, and the only factor in the discrimination of the samples according to the current cerumen/VOMs biomarkers data is cancer. These results indicate that the 27 VOMs selected by GA-PLS are promising biomarkers in cerumen for cancer diseases since the discrimination pattern shown in Fig. 1 is an excellent result when considering that the data set is of intermediate size. The 27 VOMs selected are shown in Table S4 with their respective chemical structure.

## Discussion

The cerumen analysis provides a TIC with many peaks and different profiles for each cancer type leading to the identification of a wide range of compounds (Fig. S1). A previous compendium published in 2014 presented the VOMs composition of breath, saliva, blood, milk, skin secretions, feces, and urine, but cerumen was not included mainly due to the lack of studies that explore this biomatrix^33^. However, the cerumenogram can identify 158 VOMs, which is an excellent number of compounds compared to 130 found in urine^34^ and 120 in saliva of humans^35^, using the same analytical technique, GC-MS. Therefore, cerumen is a potential source of biomarkers for many metabolomic changes, due to the wide variety of VOMs.

Using the binary data approach, 27 VOMs were selected by GA-PLS, which are 2,5-Dihydrofuran (VOM 3), 2-Butanone (VOM 5), 2-Methyl-3-buten-2-ol (VOM 6), 3-Methylhexane (VOM 10), 2-Pentanone (VOM 11), 1-Methylcyclooctene (VOM 40), 6-Methyl-7-oxabicyclo[4.1.0]heptan-2-one (VOM 42), 5-Ethyldihydro-2(3 H)-furanone (VOM 43), 6-Methyltetrahydro-2H-pyran-2-one (VOM 47), 2,5-Dimethylaniline (VOM 58), 1-Decanol (VOM 59), 6-Propyltetrahydro-2H-pyran-2-one (VOM 72), 6-Butyltetrahydro-2H-pyran-2-one (VOM 85), 3-Phenylthiophene (VOM 88), N-(3-Acetylphenyl)acetamide (VOM 99), 2,3-Dimethylquinoline (VOM 102), 1-Dodecanol (VOM 103), Dodecanoic acid (VOM 105), 1-(Decyloxy)decane (VOM 120), Eicosane (VOM 126), 6-Heptyltetrahydro-2H-pyran-2-one (VOM 128), n-Tetradecanoic acid (VOM 130), 7-Octadecanone (VOM 134), n-Octadecanoic acid (VOM 141), n-Octadecanal (VOM 142), Diisobutyl phthalate (VOM 147), and Bis(2-ethylhexyl) phthalate (VOM 157).

These data set were submitted in multivariate analysis, HCA, to visualize if the samples from cancer and control group could be discriminated. Precisely, these 27 compounds demonstrated a 100% accurate discrimination between cancer and control group samples (Fig. 1). These produced VOMs are distinct in each organism due to the cell process of cancer development in the human organism, which includes tumor-promotion inflammation and genome instability and mutation^17,36^. However, even in these different clinical cases of the patients in the cancer group, such as treatment and cancer type, these 27 VOMs selected by GA-PLS successfully discriminate all samples from cancer and control group without over-classification by ethnicity/race or gender, and they are definitely potential cancer biomarkers in cerumen.

To construct a database with a large number of individuals in healthy and cancer conditions, all variables selected by GA-PLS were maintained, although some variables, such as 1-Methylcyclooctene (VOM 40), 2,5-Dimethylaniline (VOM 58), 1-Decanol (VOM 59), 3-Phenylthiophene (VOM 88), and 1-Dodecanol (VOM 103) (Table S3), are not important for the discrimination process, and their exclusion does not change the discrimination obtained by the model until. The 1-Methylcyclooctene (VOM 40), 2,5-Dimethylaniline (VOM 58), 1-Decanol (VOM 59), 3-Phenylthiophene (VOM 88), and 1-Dodecanol (VOM 103) selected by GA-PLS are not discussed here since they are not important for discriminating the samples by multivariate analysis, but metabolites 5-Butyldihydro-2(3 H)-furanone (VOM 69) and n-Nonadecanoic acid (VOM 145) are discussed to emphasize their changes in the frequency of occurrence between cancer and control group, although they were not selected by GA-PLS. These 27 biomarkers selected by GA-PLS are from different organic class, such as organooxygen (ketones, aldehydes, furans, esters and ethers, pyrans, epoxides, and alcohols), organic acids (carboxylic acids), organonitrogen (amines and amides derivatives), organosulfur (dialkyldisulfides and thioethers), and hydrocarbons (alkanes, alkenes, benzene, and substituted derivative). Some of them are associated for the first time as expression of cancer cells.

Among the polar VOMs, the most numerous class found in cerumen were the ketones. The ketones are abundantly found in urine, breath, saliva, and tissue samples^34,35,37,38^. These compounds are produced in the human organism from many different metabolic pathways, mainly endogenous decarboxylation of acetyl-CoA, β-oxidation of fatty acids, and exogenous pathways (air contaminants, diet, smoking). The increase of some ketone bodies in the organism were associated with the growth of cancer cells^36,39,40^. As seen in Table S4, in our analysis the GA-PLS selected three ketones as cancer biomarkers in cerumen: 2-Butanone, 2-Pentanone, and 7-Octadecanone.

The 2-Butanone (VOM 5) was found in 96.2% in cancer samples, while in the control group it was found in 58% samples (38.2% difference) (Table S3). This biomarker was already detected in abnormal concentration in feces of adults with Crohn’s disease^41^, in the saliva of children with celiac disease^42^, and in the urinary profile of breast cancer patients^43^. Another ketone biomarker in cerumen, 2-Pentanone (VOM 11), when found in high levels in feces, has been associated with inflammatory bowel diseases^44^. In addition, 7-Octadecanone (VOM 134), a ketone of high molecular weight, was selected as a cancer biomarker in this biomatrix; however, this is the first time that this compound has been associated with some pathology.

In addition to the polar VOMs identified here, fourteen aldehydes were detected in cerumen analysis. Several sources, exogenous and endogenous, are responsible for a presence of aldehydes in an organism. In endogenous pathways, these carbonyl compounds arise in the body, mainly by a mechanism involving process of metabolized alcohols and reduction of hydroperoxide by cytochrome P450 as a secondary product of lipid peroxidation. For the exogenous route, tobacco smoke, the products utilized in detoxification process of tobacco, and dietary lifestyle are the main sources of aldehydes in the body^14^. Aldehydes are also commonly found in the urinary biosignature^34^, salivary profile^35^, and in gastric tissue samples^38^.

Among fourteen aldehydes identified here, only one, n-Octadecanal (VOM 142), was selected by GA-PLS as a cancer biomarker. The n-Octadecanal has been found in the plasma of patients with Sjogren-Larsson syndrome (SLS) and can also be found in feces from healthy subjects^45,46^. However, this is the first time that n-Octadecanal has been associated with cancer diseases.

Some cyclic compounds containing oxygen as a heteroatom, such as furanic, lactones, and derivatives, were identified in our analysis. In the human organism, these compounds are produced by the dehydration of monosaccharides, also as a result of the catalysis reaction of fatty acid oxidation by lipoxygenases^47^. The VOMs formed by the fatty acid peroxidation maybe liberated as a consequence of oxidative stress into the inflammatory cells by general blood circulation^48^. Otherwise, studies have suggested that these compounds have higher production in patients with colorectal cancer than in healthy individuals^47^, and high levels of these compounds are also associated with breast and lung cancer^43,49^.

The GA-PLS selected two furanic compounds as cancer biomarkers: 2,5-Dihydrofuran (VOM 3) and 5-Ethyldihydro-2(3 H)-furanone (VOM 43). The 5-Butyldihydro-2(3 H)-furanone (VOM 69) was not selected by GA-PLS; however, it seems to be a very important VOM due to its presence in 40% more samples from the cancer group than the control group (Table S3). This is the first time that 2,5-Dihydrofuran (VOM 3) has been associated with cancer diseases. In addition, the other furanic derivative compound selected as cancer biomarker in cerumen, 5-Ethyldihydro-2(3 H)-furanone (VOM 43), when presents in abnormal levels in feces, has been correlated with some gastrointestinal diseases, such as Campylobacter jejuni infection^50^.

Esters and ethers are commonly found in cerumen due to the formation processing of this biomatrix. Cerumen is formed by the combination of ceruminous glands with sebaceous glands, resulting in a mixture of fatty material and sweat secretions^51^. Thus, cerumen contains many heavy molecular weight compounds, such as wax esters, triacyclglycerols, and cholesterol^52,53^. In our bioanalytical analysis, we identified fourteen ester and ether compounds, and among them, three were selected as cancer biomarkers: 1-(Decyloxy)decane (VOM 120), Diisobutyl phthalate (VOM 147), and Bis(2-ethylhexyl) phthalate (VOM 157). This is the first time that these compounds have been indicated as biomarkers for cancer diseases. On the other hand, the phthalate compounds are known endocrine disruptors, dermal adsorption and urine secretions of these compounds has been widely study^54,55^.

In addition, the two-last classes of heterocyclic compounds, with oxygen as a heteroatom, found in cerumen samples were the Pyran and the Epoxide/Oxabicyclo compounds. We detected in cerumen samples eight Pyran and two Epoxide/Oxabicyclo compounds. The GA-PLS selected four Pyran compounds as cancer biomarkers: 6-Methyltetrahydro-2H-pyran-2-one (VOM 47), 6-Propyltetrahydro-2H-pyran-2-one (VOM 72), 6-Butyltetrahydro-2H-pyran-2-one (VOM 85), and 6-Heptyltetrahydro-2H-pyran-2-one (VOM 128). In addition, one Epoxide/Oxabicyclo compound was selected: 6-Methyl-7-oxabicyclo[4.1.0]heptan-2-one (VOM 42).

However, only 6-Methyltetrahydro-2H-pyran-2-one has already been detected in abnormal conditions in feces of patients with colorectal cancer^56^. The other three Pyran compounds related here are being described as cancer biomarkers for the first time. The epoxide compounds are produced in the human organism by the isoprene metabolization in liver microsomes by cytochrome P450 (CYP2E1 and CYPB6) to mono and di-epoxides compounds^57,58^. The Epoxide selected here as cancer biomarker in cerumen is also being related for the first time as an expression of cancer cells.

The analysis of cerumen identified thirteen alcohols and derivatives. Alcohols have exogenous and endogenous routes that can explain their presence in the human organism. In the endogenous ways, alcohols are a secondary product of lipid peroxidation, and low-weight alcohols are produced in pyruvate metabolism by intestinal bacteria^37,59^. For the exogenous routes, alcohols are release in the gastrointestinal tract into the blood, following enzyme metabolization (such as alcohol dehydrogenases), concomitantly with the reduction of nicotinamide adenine dinucleotide (NAD^+^ to NADH), and by cytochrome P450 (CYP2E1)^14^.

Alcohols have many routes of body-scape, mainly through urine, sweat, feces, breath, saliva, and breast milk^17^. Three alcohols were selected by GA-PLS as cancer biomarker in cerumen: 2-Methyl-3-buten-2-ol (VOM 6), 1-Decanol (VOM 59), and 1-Dodecanol (VOM 103). However, the 1-Decanol only appeared in 10% of the control samples, while this compound was not found in the cancer group (Table S3). The 1-Dodecanol only changed 0.6% between cancer and control group (Table S3). The 2-Methyl-3-buten-2-ol was the main biomarker found in this class, and it is being reported here as a cancer biomarker for the first time.

We identified fourteen carboxylic acids in the cerumen biomatrix. Carboxylic acids are produced in the organism and mainly released through oxidation of cytotoxic aldehydes dehydrogenase (ALDH) enzymes^60^. Studies identify volatile organic acids as important intermediates in different biological processes usually due to bacterial activity, such as degradation of carbohydrates in the intestine by bacterial anaerobic process^61^. These organic compounds are commonly found in urine^34^, saliva^35^, and human tissue^38^.

This analysis separated three organic acids as cancer biomarker in cerumen by GA-PLS. They are Dodecanoic acid (VOM 105), n-Tetradecanoic acid (VOM 130), n-Octadecanoic acid (VOM 141). The n-Nonadecanoic acid (VOM 145) was not selected by GA-PLS; however, this compound appears 53.4% more in cancer group than in control group samples (Table S3). These compounds, when present in abnormal concentration in many biomatrices, are widely associated with advanced or immature cancer pathology. Dodecanoic acid (VOM 105) has been found at high levels in saliva of patients with oral squamous cell carcinoma (OSCC), in patients with oral leukemia (OLK)^62^, colorectal patients^56^, and patients with metastatic melanoma^63^.

The n-Tetradecanoic acid (VOM 130), when detected in high levels in urine, has been associated with OLK and OSCC^62^, and detected in abnormal concentration in blood samples for an oesophageal cancer group^64^, and in feces and urine of colorectal patients^65,66^. In addition, high levels of n-Octadecanoic acid (VOM 141) has already been associated as a biomarker for breast cancer in blood analysis^67^, and in feces for colorectal cancer^24^. Finally, the n-Nonadecanoic acid (VOM 145) has also been detected in high levels in feces of patients with colorectal cancer^68^.

We have identified seventeen organic compounds in cerumen that has nitrogen as a heteroatom. Organonitrogen compounds, such as pyrrole, pyridine, amides, and derivatives were found in cerumen, and they can be related as natural products of the organism or associated to pollutants of exogenous sources (*e*.*g*. diet, air pollution, and cigarette smoke)^69,70^. Furthermore, some higher volatile organonitrogen emission from tissue samples have been associated with gastric, and lung tissue cancer^38,71^. In this analysis, three organonitrogen compounds were selected by GA-PLS as a cancer biomarker in cerumen: 2.5-Dimethylaniline (VOM 58), N-(3-Acetylphenyl)acetamide (VOM 99), and 2,3-Dimethylquinoline (VOM 102). This is the first time that these compounds have been found as an expression of some pathology.

The incomplete metabolism of cysteine and methionine by the transamination pathway are responsible for the expression of volatile organosulfur^72^. We detected three organosulfur compounds in the cerumen samples, among them, the Dimethyl disulfide (VOM 16), which was produced through the oxidation of methanethiol, also by gram-negative bacteria^72,73^. Interestingly, Dimethyl disulfide (VOM 16) has been detected as a potential cancer biomarker in many biomatrices, especially urine^49^, but in cerumen was not selected as a cancer biomarker. In cerumen only the 3-Phenylthiophene (VOM 88), of this class, was selected as a biomarker by GA-PLS; however, this compound is present only 2.5% more in cancer than in control group samples (Table S3).

We detected twenty-four hydrocarbons, the second most present class in cerumen samples. Many production routs of hydrocarbons have been connected to the presence of reactive oxygen species, due to oxygen free radicals that probably escape from mitochondria into a cytoplasm^37^. This analysis selected three hydrocarbons as cancer biomarkers: 3-Methylhexane (VOM 10), 1-Methylcyclooctene (VOM 40), and Eicosane (VOM 126). Eicosane, which was probably produced by peroxidation of polyunsaturated fatty acids (PUFAs), *e*.*g*. *linoleic acid*, present in the cell membrane^74^, has already been detected in saliva and feces of healthy subjects^33,75^. In addition, 3-Methylhexane, a result of peroxidation of PUFAs^76^, is related for the first time in the human organism. Thus, these hydrocarbons are found exclusively in cerumen as valuable cancer biomarkers.

In summary, we developed a new analytic approach to identify cancer in humans with some advances to another methods. The main merits of cerumen sampling over other non-invasive biomatrix include easy sample collection, painless and no discomfort or embarrassment associated with other biomatrices (such as blood, urine, and feces tests), no need for treatment or preconcentration of the sample, less inclined to contamination, and not liable to blood contamination of samples^25^.

In addition, cerumen was the first biomatrices to achieve a 100% efficiency in the discrimination between samples from cancer and control group, and the only limitation is the inability to discriminate between cancer types that would require further specific screening process^77,78^. However, it is worth emphasizing that, given its high reliability, accuracy, simplicity, and low cost, this test for cancer and other diseases using cerumen – here called for the first time as Cerumenogram – could be used frequently as a preceding diagnosis to be applied before the use of specific diagnostic methods for each type of cancer, which are much more expensive and still very rare.

## Conclusion

Cerumen is a fingerprint of both polar and nonpolar substances excreted by biochemical reactions, and cancer cells produce different substance than healthy cells. These differences in chemical composition can be monitored to determine cancer early, since cerumen is continuously excreted. This new analytical test development obtained 100% efficiency in the discrimination of all samples used in this work, separating the cancer from the control group samples. The Cerumenogram is performed in a total time of around 3 hours, with an estimated analysis cost of US$ 50/per sample. This means that the analysis of VOMs in cerumen is a simple, fast, and cheap way to identify cancer, with the highest accuracy possible for a human that suffers from cancer. Furthermore, cerumen presents many advantages, such as painless collection and no liability of external contamination. This new method will allow a number of biomarkers identified for cancer diseases, 27 VOMs, to be monitored in an emergency or routine test, substantially reducing deaths from these diseases. The trend is that, from the encouraging data obtained here, soon, a so-called Cerumenogram will be a diagnostic test as common as a blood count is today, making it possible to save lives that could be eradicated by one of the deadliest diseases.

## Methods

### Test population: Patients and sample collection

Cerumen samples from 102 volunteers were collected at oncology unit of the Clinical Hospital, Federal University of Goiás (HC/UFG – Goiás, Brazil). Samples were collected from volunteers’ ears using a metallic curette and transferred in Eppendorf tubes, which were closed and stored in a freezer at −20 °C. All analyses were carried out a maximum of 7 days after the collect. The patients were divided into two groups: control group (Healthy subjects; cancer free, n = 50, age range 2–65 years old, 29 males and 21 females), and cancer group (n = 52, age range 33–83 years old, 25 males and 27 females).

### Ethics committee approval

All volunteers who agreed to participate in this study signed an informed consent after the approval of local ethics committee at the Federal University of Goiás (#57880516.9.0000.5083). A questionnaire about their medical history was applied with the aimed of identifying and eliminating metabolites from medications, licit drugs, and treatment of past diseases that can show VOMs not coming from the cancer diseases or from the patient’s health conditions. Every step in this study was strictly conducted by following the Declaration of Helsinki. The main information extracted from the volunteers’ questionnaires are summarized in Table S2.

### VOMs analysis

Cerumen samples were analyzed according to the recent method created in our research group^29^. In this way, 20 mg of each cerumen sample collected was weighed into 20 mL GC headspace vials and 0.2 µL of 3-methylcyclohexanone (Sigma-Aldrich, Saint Louis, MO, USA) was added as IS. In addition, gas-tight polytetrafluoroethylene (PTFE)-lined rubber septum caps were used to seal the vials. Then, the analyses were carried out by HS/GC-MS, applying all steps pre-established for GC-MS-based metabolomics, such as baseline correction, noise reduction, retention time alignment, and data-normalization^79,80^.

### HS/GC-MS equipment

The cerumen sample analyses were conducted using a Shimadzu GCMS-QP2010 Ultra system and a Shimadzu AOC-5000 headspace analyzer (Shimadzu, Japan). The system uses a 2500 µL gas-tight syringe, a VT32–20 tray for 20 mL standard vials (PAL System, Zwingen, Switzerland) with a preheating module LHS0 Combi Pal with heating time and control of temperature (PAL System, Zwingen, Switzerland).

### Headspace

Headspace sampler parameters were configured at: fill volume (2500 µL), fill speed (100 µL s^−1^), injection volume (2500 µL), injection speed (1 mL s^−1^), syringe temperature (150 °C), pre-warm time (10 min), equilibration time (3 min), syringe flushing time (5 min), re-flush time (5 ms), and post-flush time (5 ms). The preheating module parameters were set at: agitation speed (500 rpm), agitation on time (5 s), agitation off time (3 s), incubation temperature (160 °C), time of incubation (60 min).

### Gas chromatography

VOMs were eluted in an analytical capillary column NST-100-ms (25 m × 0.25 mm i.d. × 0.3 µm film thickness) (NST, São Paulo, Brazil) with a polyethylene glycol high-polarity stationary phase. The injector was operated at 250 °C in the splitless mode applied with high purity helium (99.999% - 5.0, Helium, Air Liquide) as a carrier gas with a constant flow rate of 1.36 mL min^−1^ with a linear velocity for the carrier gas of 45.8 cm s^−1^. The oven temperature programming was set at: elution start in 30 °C (with isothermal heating for 5 min), a 2 °C min^−1^ gradient up until 45 °C (held 5 min), followed by another increase at 2 °C min^−1^ to 50 °C (held 5 min), another increase at 2 °C min^−1^ to until 120 °C, and another with 6 °C min^−1^ to 200 °C (held 5 min) ending at 5 °C min^−1^ to 250 °C (held 10 min), for a total of GC run time of 98.33 min.

### Mass spectrometry

MS spectra of VOMs were acquired by electron ionization (EI) mode at 70 eV. The star cut-off time for MS recording was 0 min. Data acquisition was performed in full scan mode from 40 to 500 *m/z* with a scan time of 0.3 s and a scan speed of 1666 u s^−1^. The cerumen VOMs were confirmed by comparing their MS patterns with those of valid standards (ST) run in the same GC conditions, and by NIST11s Mass Spectral Library. Only compounds with more than 80% probability of a match to NIST11s library standards were considered. Finally, all the VOMs chromatographic peaks were confirmed by their respective retention time relative to the IS.

### Statistical methods

The raw data generated in our analysis provides a total of 158 observations (chromatographic peaks) extracted from the test groups. A binary data model was constructed, where the 158 variables detected were transformed into binary output express as 1 for presence and 0 for absence, resulting in a data matrix of 102 rows representing the earwax samples by 158 columns for the VOMs.

### Data treatment

A variable selection procedure using the GA-PLS results in 27 selected variables. The GA-PLS parameters were set as: population size of 100, window width at 1, maximum number of variables in each population of 100, convergence probability of 50%, mutation probability of 0.5%, maximum number of generations of 35, and contiguous cross-validation. HCA analyses using Ward agglomeration method were run applying Hamming distances as proximity measures of the binary data. GA-PLS was run according to PLS Toolbox 7.9 (Eigenvector Research Inc., Manson, WA, USA) algorithm using Matlab 2014b (MathWorks, Natick, MA, USA). The e1071 package was used to calculate Hamming distances under R version 3.5.1 (R Foundation for Statistical Computing, http://www.R-project.org)^81^.

### Materials & correspondence

All data needed to interpret conclusions herein are presented in the paper and/or the Supplementary Information. Additional data related to this paper may be requested from the corresponding authors (J.M.G.B. and N.R.A.F.). Only the questionnaires filled out by the volunteers during the collection will not be available, due to the signature of their identity preservation agreement.

## Supplementary information

Supplementary information
