# Supplementary material for: Cerumenogram: a new frontier in cancer diagnosis in humans
Source: Sci Rep. 2019 Aug 13;9:11722. doi: 10.1038/s41598-019-48121-4 (PMC6692389; doi:10.1038/s41598-019-48121-4)
Supplement: Supplementary file 1 — Supplementary information [file 41598_2019_48121_MOESM1_ESM.docx]

Supplementary Information for

**Cerumenogram: a new frontier in cancer diagnosis in humans**

João Marcos G. Barbosa, Naiara Z. Pereira, Lurian C. David_,_ Camilla G. de Oliveira, Marina F. G. Soares, Melissa Ameloti G. Avelino, Anselmo E. de Oliveira, Engy Shokry, Nelson R. Antoniosi Filho

Corresponding to :[joaomarcosquim.ufg@outlook.com](mailto:joaomarcosquim.ufg@outlook.com) (João Marcos G. Barbosa) and [nelsonroberto@ufg.br](mailto:nelsonroberto@ufg.br)**(**Nelson R. Antoniosi Filho).

**This file includes:**

Figure S1-S6

Tables S1-S4


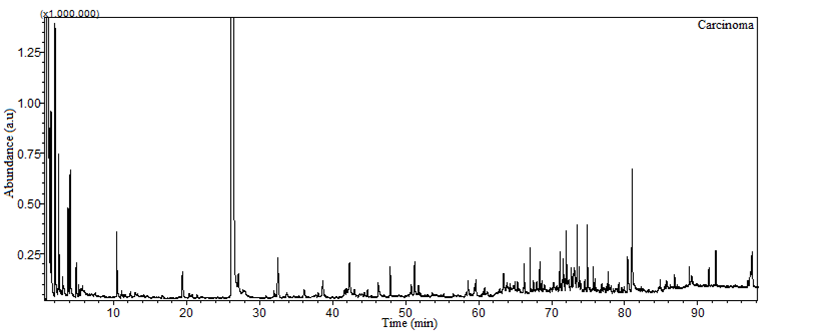

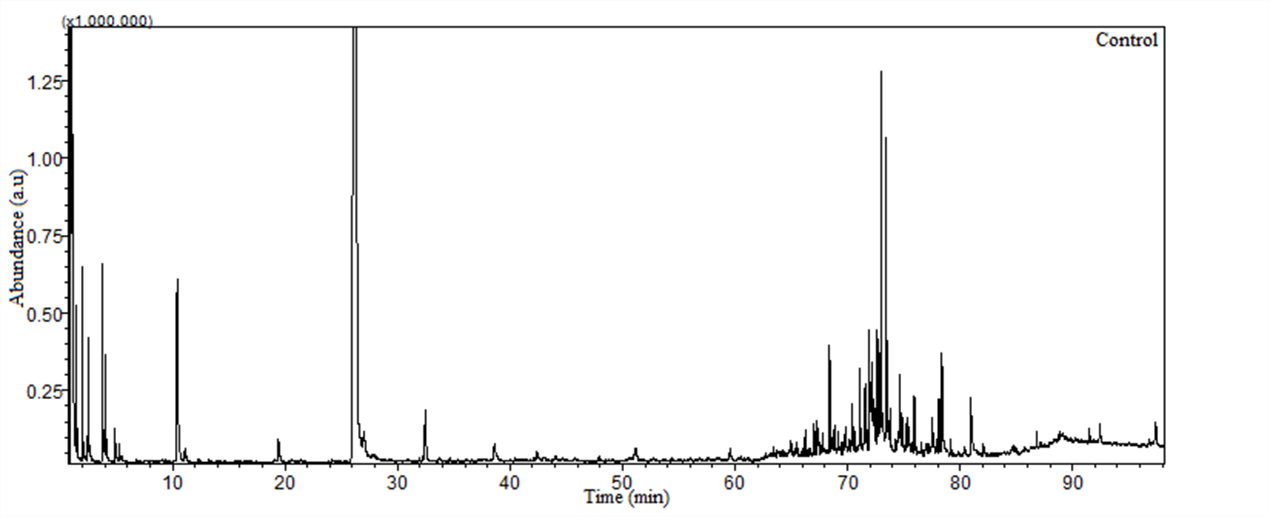


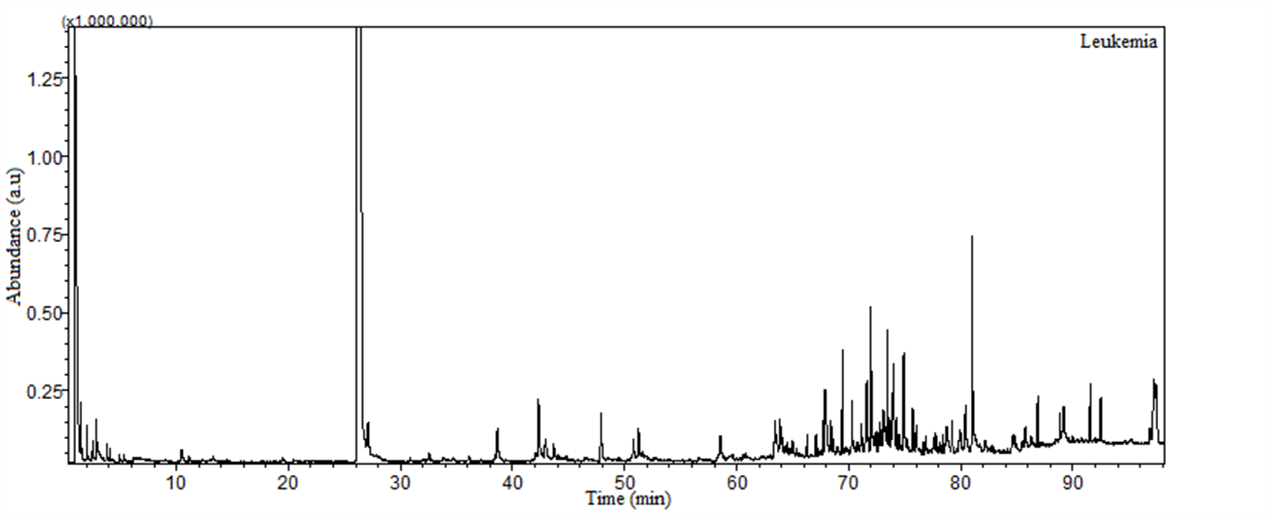


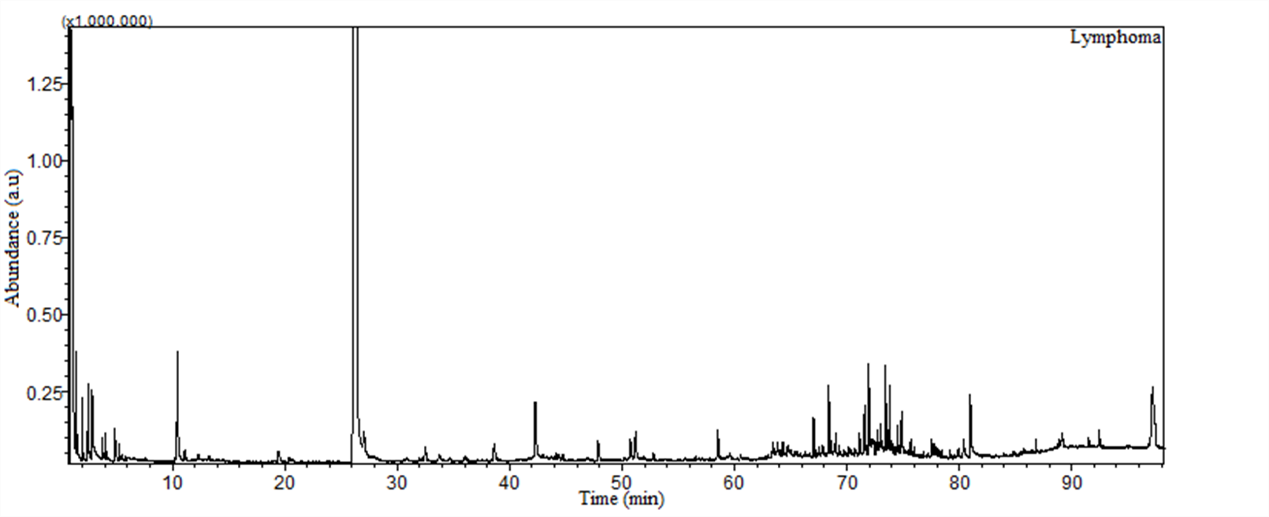


**Figure S1.** Fingerprint signals of VOM cerumen profile of healthy subjects (control) contrasted with cancer patients (Carcinoma, leukemia, and lymphoma). Total Ion Chromatograms (TIC) of cerumen samples were obtained by Headspace/Gas Chromatography-Mass Spectrometry (HS/GC-MS). The GC run was performed with addition of 3-Methylcyclohexanone (Average Retention Time: 26.332 min) as internal standard (IS).


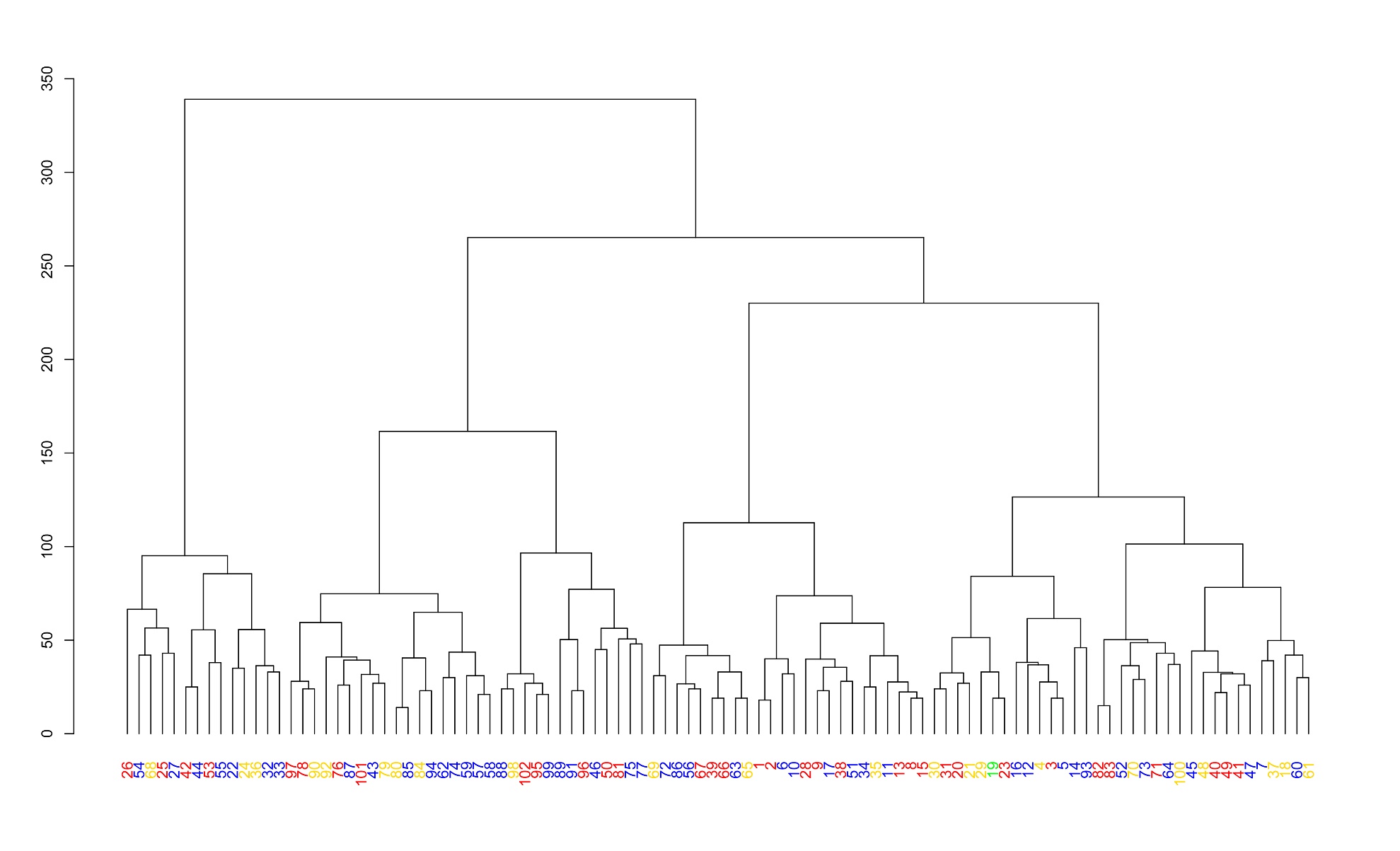


**Figure S2**. Hierarchical Cluster Analysis (HCA) using all the 158 Volatile Organic Metabolites (VOMs) identified to evaluate the influence of ethnicity/race. Cerumen samples: Blue = Multiracial people; Yellow = White people; Green = Indigenous people; Red = Black people.


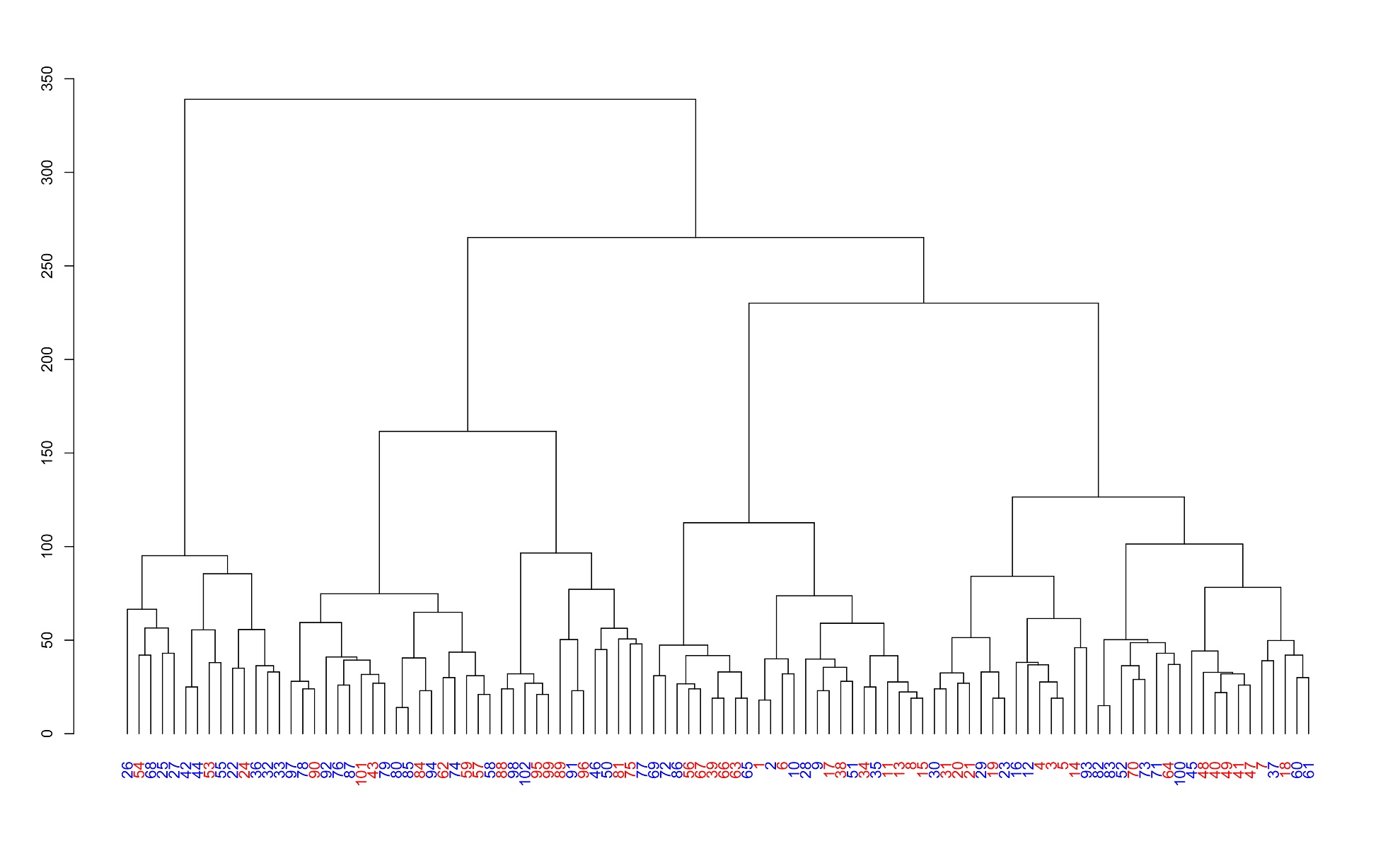


**Figure S3.** Hierarchical Cluster Analysis (HCA) using all the 158 Volatile Organic Metabolites (VOMs) identified to evaluate the gender influence. Cerumen samples: Blue = Male; Red = Female.


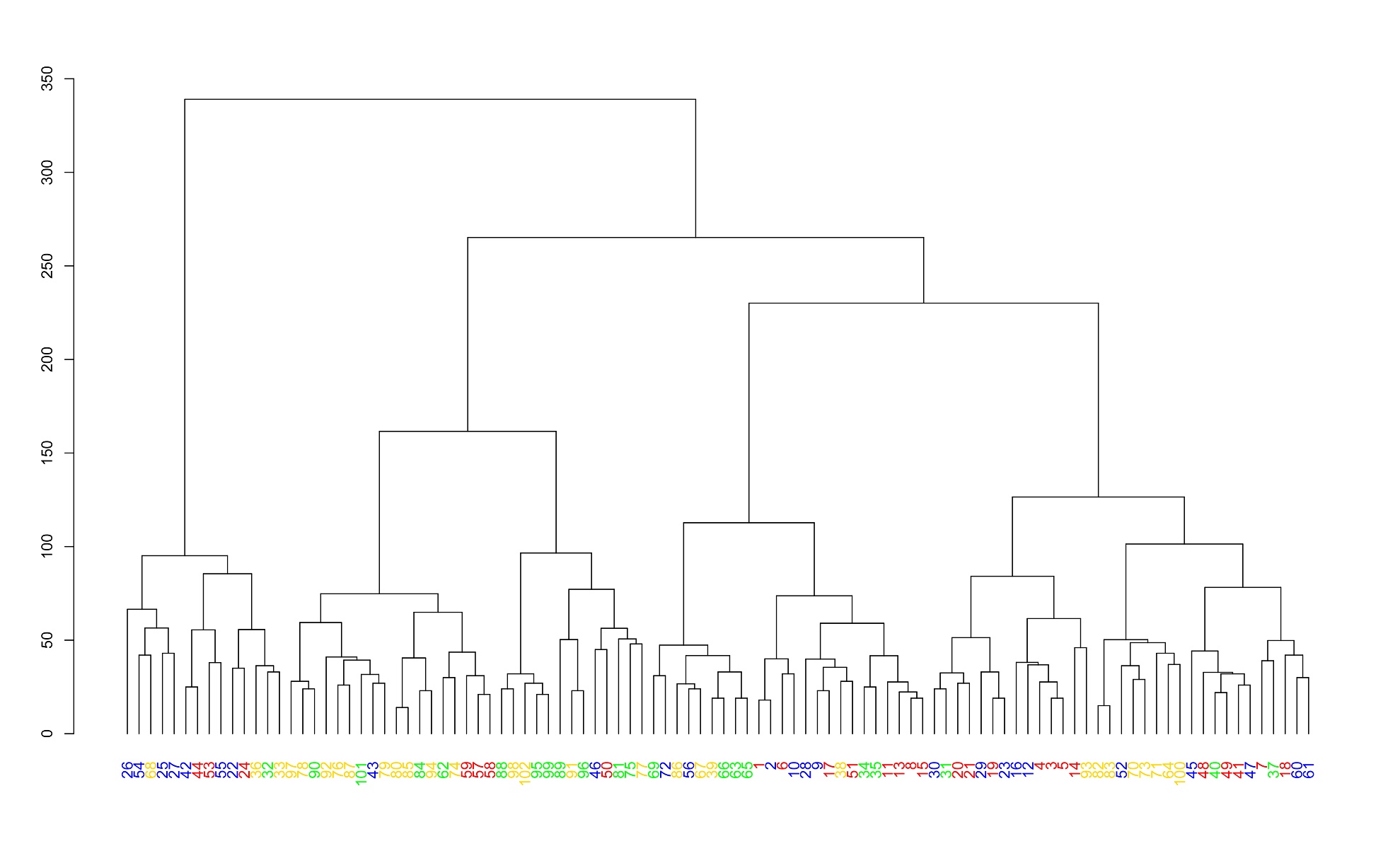


**Figure S4**. Hierarchical Cluster Analysis (HCA) using all the 158 Volatile Organic Metabolites (VOMs) identified to evaluate the gender influence with regard to the healthy/cancer condition. Cerumen samples: Blue = Cancer-Male; Yellow = Healthy-Male; Green = Healthy-Female; Red = Cancer-Female.


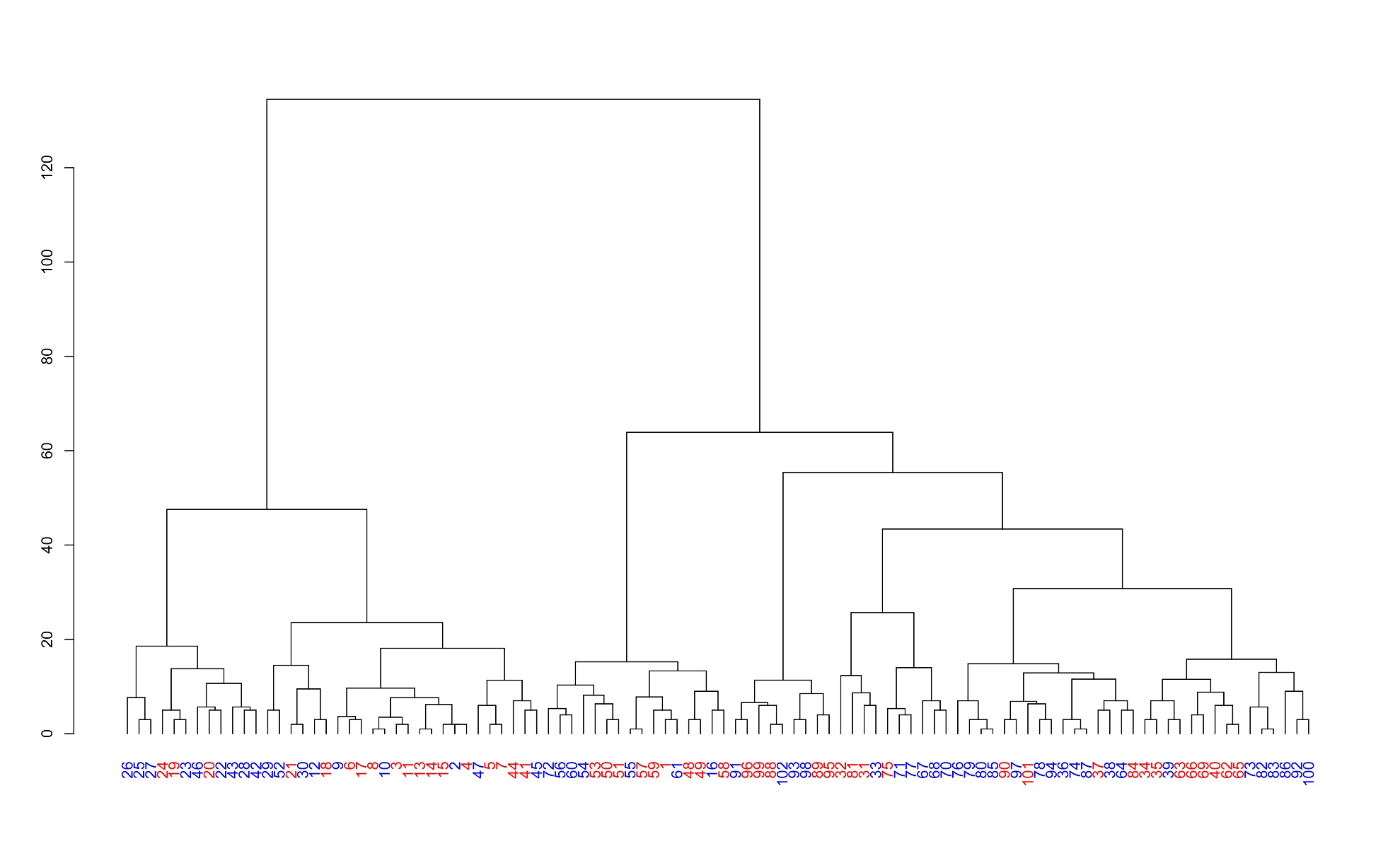


**Figure S5.** Hierarchical Cluster Analysis (HCA) using the 27 Volatile Organic Metabolites (VOMs) selected as potential cancer biomarkers to evaluate the gender influence. Cerumen samples: Blue = Male; Red = Female.


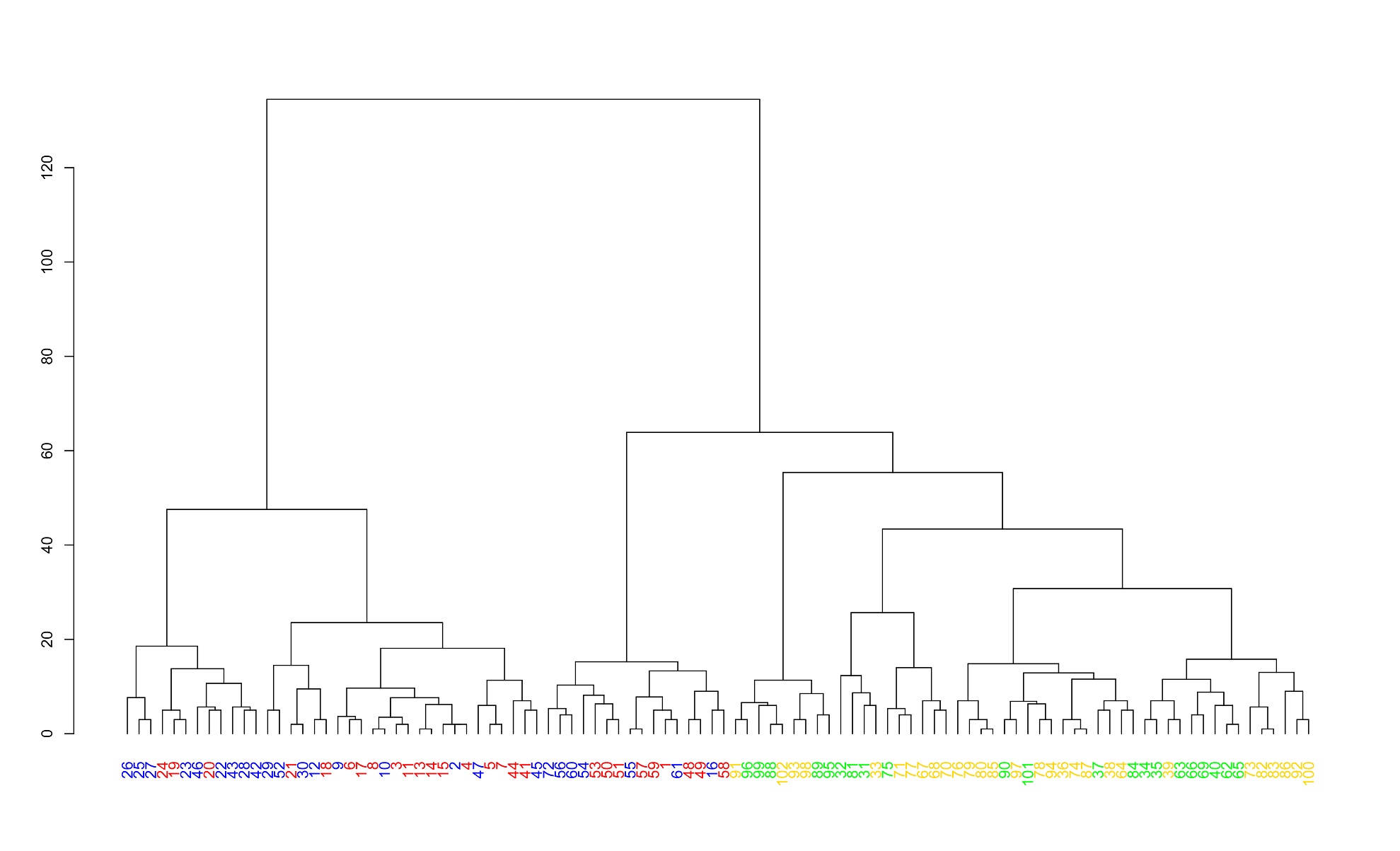


**Figure S6**. Hierarchical Cluster Analysis (HCA) using the 27 Volatile Organic Metabolites (VOMs) selected as potential cancer biomarkers to evaluate the gender influence with regard to the healthy/cancer condition. Blue = Cancer-Male; Yellow = Healthy-Male; Green = Healthy-Female; Red = Cancer-Female.

**Table S1.** Cerumen samples’ information about the order of analysis, codes, date of collection, gender, cancer type and treatment of the volunteers. The colors of the numbers represent the colors of the samples in Figure 1. Red = cancer; Blue = healthy.

| Order of Samples' Analyses | Samples Codes | Date of collection | Gender^A^ | Cancer^B^ | Cancer Type/Healthy^C^ | Treatment^D^ |
| --- | --- | --- | --- | --- | --- | --- |
| 1 | A1 | **03/19/2018** | F | Y | CA | C |
| 2 | A2 | 03/19/2018 | M | Y | CA | C |
| 3 | A3 | 03/19/2018 | F | Y | CA | C |
| 4 | A4 | 03/19/2018 | F | Y | CA | C |
| 5 | A5 | 03/19/2018 | F | Y | CA | R |
| 6 | A6 | 03/19/2018 | F | Y | CA | N |
| 7 | A7 | 03/19/2018 | F | Y | CA | N |
| 8 | A8 | 03/19/2018 | F | Y | CA | C |
| 9 | A9 | 03/19/2018 | M | Y | CA | N |
| 10 | A10 | 03/19/2018 | M | Y | CA | N |
| 11 | A11 | 03/19/2018 | F | Y | CA | C |
| 12 | B1 | **03/20/2018** | M | Y | CA | N |
| 13 | B2 | 03/20/2018 | F | Y | CA | N |
| 14 | B3 | 03/20/2018 | F | Y | CA | N |
| 15 | B4 | 03/20/2018 | F | Y | CA | C |
| 16 | B5 | 03/20/2018 | M | Y | CA | N |
| 17 | B6 | 03/20/2018 | F | Y | CA | N |
| 18 | B7 | 03/20/2018 | F | Y | CA | N |
| 19 | B8 | 03/20/2018 | F | Y | CA | C |
| 20 | C1 | **03/26/2018** | F | Y | CA | N |
| 21 | C2 | 03/26/2018 | F | Y | CA | C |
| 22 | C3 | 03/26/2018 | M | Y | CA | C/R |
| 23 | C4 | 03/26/2018 | M | Y | CA | C/R |
| 24 | C6 | 03/26/2018 | F | Y | LK | C |
| 25 | C7 | 03/26/2018 | M | Y | LK | N |
| 26 | C8 | 03/26/2018 | M | Y | LK | N |
| 27 | C9 | 03/26/2018 | M | Y | LK | N |
| 28 | C10 | 03/26/2018 | M | Y | LK | N |
| 29 | C11 | 03/26/2018 | M | Y | LK | C |
| 30 | C12 | 03/26/2018 | M | Y | LK | N |
| 31 | D1 | 03/26/2018 | F | N | H | N |
| 32 | D2 | 03/26/2018 | F | N | H | N |
| 33 | D3 | 03/26/2018 | M | N | H | N |
| 34 | E1 | 03/26/2018 | F | N | H | N |
| 35 | E2 | 03/26/2018 | F | N | H | N |
| 36 | E3 | 03/26/2018 | M | N | H | N |
| 37 | E4 | 03/26/2018 | F | N | H | N |
| 38 | E5 | 03/26/2018 | M | N | H | N |
| 39 | E6 | 03/26/2018 | M | N | H | N |
| 40 | E7 | 03/26/2018 | F | N | H | N |
| 41 | F1 | 03/26/2018 | F | Y | LP | C |
| 42 | F2 | 03/26/2018 | M | Y | LP | C |
| 43 | F3 | 03/26/2018 | M | Y | LP | C |
| 44 | F4 | 03/26/2018 | F | Y | CA | N |
| 45 | F5 | 03/26/2018 | M | Y | LP | C |
| 46 | F6 | 03/26/2018 | M | Y | LP | C |
| 47 | F7 | 03/26/2018 | M | Y | CA | C |
| 48 | F8 | 03/26/2018 | F | Y | CA | C/R |
| 49 | F9 | 03/26/2018 | F | Y | LP | C |
| 50 | F10 | 03/26/2018 | F | Y | LP | N |
| 51 | F11 | 03/26/2018 | F | Y | LP | N |
| 52 | F12 | 03/26/2018 | M | Y | LP | N |
| 53 | F13 | 03/26/2018 | F | Y | CA | N |
| 54 | F14 | 03/26/2018 | M | Y | LK | C/R |
| 55 | F15 | 03/26/2018 | M | Y | LK | C |
| 56 | F16 | 03/26/2018 | M | Y | LK | C |
| 57 | F17 | 03/26/2018 | F | Y | LK | C |
| 58 | F18 | 03/26/2018 | F | Y | LK | C/R |
| 59 | F19 | 03/26/2018 | F | Y | LK | C/R |
| 60 | F20 | 03/26/2018 | M | Y | LP | N |
| 61 | F21 | 03/26/2018 | M | Y | LP | N |
| 62 | G1 | **03/29/2018** | F | N | H | N |
| 63 | G2 | 03/29/2018 | F | N | H | N |
| 64 | G3 | 03/29/2018 | M | N | H | N |
| 65 | G4 | 03/29/2018 | F | N | H | N |
| 66 | G5 | 03/29/2018 | F | N | H | N |
| 67 | G6 | 03/29/2018 | M | N | H | N |
| 68 | G7 | 03/29/2018 | M | N | H | N |
| 69 | G8 | 03/29/2018 | F | N | H | N |
| 70 | G9 | 03/29/2018 | M | N | H | N |
| 71 | G10 | 03/29/2018 | M | N | H | N |
| 72 | H1 | **04/06/2018** | M | Y | CA | C/R |
| 73 | H2 | 04/06/2018 | M | N | H | N |
| 74 | H3 | 04/06/2018 | M | N | H | N |
| 75 | H4 | 04/06/2018 | F | N | H | N |
| 76 | H5 | 04/06/2018 | M | N | H | N |
| 77 | H6 | 04/06/2018 | M | N | H | N |
| 78 | I1 | 04/06/2018 | M | N | H | N |
| 79 | I2 | 04/06/2018 | M | N | H | N |
| 80 | I3 | 04/06/2018 | M | N | H | N |
| 81 | I4 | 04/06/2018 | F | N | H | N |
| 82 | I5 | 04/06/2018 | M | N | H | N |
| 83 | I6 | 04/06/2018 | M | N | H | N |
| 84 | I7 | 04/06/2018 | F | N | H | N |
| 85 | I8 | 04/06/2018 | M | N | H | N |
| 86 | I9 | 04/06/2018 | M | N | H | N |
| 87 | I10 | 04/06/2018 | M | N | H | N |
| 88 | I11 | 04/06/2018 | F | N | H | N |
| 89 | I12 | 04/06/2018 | F | N | H | N |
| 90 | I13 | 04/06/2018 | F | N | H | N |
| 91 | I14 | 04/06/2018 | M | N | H | N |
| 92 | I15 | 04/06/2018 | M | N | H | N |
| 93 | I16 | 04/06/2018 | M | N | H | N |
| 94 | I17a | 04/06/2018 | M | N | H | N |
| 95 | I17b | 04/06/2018 | F | N | H | N |
| 96 | I18 | 04/06/2018 | F | N | H | N |
| 97 | I19 | 04/06/2018 | M | N | H | N |
| 98 | I20 | 04/06/2018 | M | N | H | N |
| 99 | I21 | 04/06/2018 | F | N | H | N |
| 100 | I27 | 04/06/2018 | M | N | H | N |
| 101 | I28 | 04/06/2018 | F | N | H | N |
| 102 | I29 | 04/06/2018 | M | N | H | N |

^A^Gender: M = Male, F=Female. ^B^Cancer: Y=Yes, N=No. ^C^Cancer Type/Healthy: CA = Carcinoma, LK = Leukemia, LP = Lymphoma, H=Healthy. Treatment: C=Chemotherapy, R=Radiotherapy, C/R=Chemotherapy and Radiotherapy, N=None.

Table S2. Information about volunteers’ medical history, lifestyle, and nutritional habits. All information was extracted from the questionnaires filled out by the volunteers during the sample collection.

| **Characteristics** | Control Group | **Histological Cancer Type** | | | Total Cancer Group |
| --- | --- | --- | --- | --- | --- |
|  |  | Lymphoma | Carcinoma | Leukemia |  |
| Number of subjects, n (%) | 50 (100.0%) | 11 (21.15%) | 28 (53.85%) | 13 (25.0%) | 52 (100.0%) |
| **Age range, n (%)** |  | | | | |
| Children and Young People (1- 17 years) | 5 (10%) | 0 | 0 | 0 | 0 |
| Young Adults (18 -35 years) | 17 (34%) | 4 (36.4%) | 3 (10.7%) | 0 | 7 (13.5%) |
| Middle age Adults (36 - 55 years) | 23 (46%) | 4 (36.4%) | 10 (35.7%) | 2 (15.4%) | 16 (30.8%) |
| Older Adults (> 55 years) | 5 (10%) | 3 (27.2%) | 15 (53.6%) | 11(84.6%) | 29 (55.7%) |
| **Sex, n (%)** |  |  |  |  |  |
| Males | 29 (58%) | 8 (72.7) | 9 (32.1%) | 8 (61.5%) | 25 (48%) |
| Females | 21 (42%) | 3 (27.3%) | 19 (67.9%) | 5 (38.5%) | 27 (52%) |
| **Ethnicity, n (%)** |  |  |  |  |  |
| Blacks | 17 (34%) | 4 (36.4%) | 10 (35.7%) | 3 (23.1%) | 17 (32.7%) |
| Whites | 14 (28%) | 1 (9.1%) | 4 (14.3%) | 3 (23.1%) | 8 (15.4%) |
| Multiracials | 19 (38%) | 6 (54.5) | 13 (46.4%) | 7 (53.8%) | 26 (50%) |
| Indigenous | 0 | 0 | 1 (3.6%) | 0 | 1 (1.9%) |
| **Cancer History** |  | | | | |
| **1.** First diagnostic, n (%) |  |  |  |  |  |
| Between 0 and 6 months | 0 | 3 (27.3%) | 3 (27.3%) | 0 | 6 (11.6%) |
| Between 7 and 11 months | 0 | 0 | 8 (72.7%) | 2 (15.4%) | 10 (19.2%) |
| Between 1 and 5 years | 0 | 6 (54.5%) | 11 (39.3%) | 6 (46.1%) | 23 (44.2%) |
| More than 5 years | 0 | 2 (18.2%) | 6 (21.4%) | 5 (38.5%) | 13 (25%) |
| **2.** Cancer treatment, n (%) |  |  |  |  |  |
| Chemotherapy | 0 | 6 (54.5%) | 10 (35.7%) | 5 (38.5%) | 21 (40.4%) |
| Radiotherapy | 0 | 0 | 1 (3.6%) | 0 | 1 (1.9%) |
| Both (Chemotherapy and Radiotherapy) | 0 | 0 | 4 (14.3%) | 3 (23%) | 7 (13.5%) |
| None | 0 | 5 (45.5%) | 13 (46.4%) | 5 (38.5%) | 23 (44.2%) |
| **3**. Metastasis, n (%) | 0 | 1 (9.1%) | 4 (14.3%) | 0 | 5 (9.6%) |
| **Surgical historic** |  |  |  |  |  |
| **1.** Number of surgeries, n (%) |  |  |  |  |  |
| None | 44 (88%) | 8 (72.7%) | 18 (64.3%) | (84.6%) | 37 (71.1%) |
| From 1 to 5 | 5 (10%) | 3 (27.3%) | 9 (32.1%) | 2 (15.4%) | 14 (26.9%) |
| More than 5 | 1 (2%) | 0 | 1 (3.6%) | 0 | 1 (1.9%) |
| **Smoking Status, n (%)** |  |  |  |  |  |
| Active Smoker | 4 (8%) | 2 (18.2%) | 3 (10.7%) | 4 (30.8%) | 9 (17.3%) |
| Non-Smoker | 28 (56%) | 3 (27.3%) | 16 (57.1%) | 7 (53.8%) | 26 (50%) |
| Passive Smoker | 13 (26%) | 0 | 3 (10.7%) | 0 | 3 (5.8%) |
| Ex-Smoker | 5 (10%) | 6 (54.5%) | 6 (21.5%) | 2 (15.4%) | 14 (26.9%) |
| **Alcohol Consumers, n (%)** |  |  |  |  |  |
| Beer | 21 (42%) | 6 (54.5%) | 11 (39.3%) | 7 (53.8%) | 24 (46.1%) |
| Wines | 14 (28%) | 2 (18.2%) | 5 (17.9%) | 2 (15.4%) | 9 (17.3%) |
| Distilled beverages | 5 (10%) | 1 (9.1%) | 4 (14.3%) | 4 (30.8%) | 9 (17.3%) |
| **Co-morbidities, n (%)** |  |  |  |  |  |
| **1.** Cardiovascular diseases |  |  |  |  |  |
| 1.1 Hypertension | 7 (14%) | 2 (18.2 %) | 6 (21.4%) | 3 (23.1%) | 11 (21.1%) |
| 1.2 Hypotension | 2 (4%) | 1 (9.1%) | 2 (7.1%) | 1 (7.7%) | 4 (7.7%) |
| **2.** Diabetes *mellitus* |  |  |  |  |  |
| 2.1 Type I | 1 (2%) | 0 | 0 | 0 | 0 |
| 2.2 Type II | 0 | 0 | 1 (3.6%) | 1 (7.7%) | 2 (3.8%) |
| **3.** Arthritis |  |  |  |  |  |
| 3.1 Osteoarthritis | 0 | 2 (18.2 %) | 2 (7.1%) | 0 | 4 (7.7%) |
| **4.** Endocrine disorders | 1 (2%) | 0 | 2 (7.1%) | 1 (7.7%) | 3 (5.8%) |
| **5.** Gastritis | 0 | 0 | 1 (3.6%) | 1 (7.7%) | 2 (3.8%) |
| **6.** Hepatitis B | 0 | 1 (9.1%) | 0 | 1 (7.7%) | 2 (3.8%) |
| **7.** Renal insufficiency | 0 | 0 | 1 (3.6%) | 2 (15.4%) | 3 (5.8%) |
| **8.** Asthma, n (%) | 1 (2%) | 0 | 0 | 0 |  |
| **9.** Hypercholesterolemia | 1 (2%) | 0 | 1 (3.6%) | 0 | 1 (1.9%) |
| **Other treatments** |  |  |  |  |  |
| **1.** Antidepressive drugs |  |  |  |  |  |
| Sertraline (Zoloft) | 2 (4%) | 0 | 1 (3.6%) | 0 | 1 (1.9%) |
| **2.** Antihypertensive drugs |  |  |  |  |  |
| Captopril | 4 (8%) | 1 (9.1%) | 1 (3.6%) | 0 | 2 (3.8%) |
| Alprazolam | 2 (4%) | 0 | 1 (3.6%) | 0 | 1 (1.9%) |
| Losartan | 1 (2%) | 2 (18.2 %) | 2 (7.1%) | 1 (7.7%) | 5 (9.6%) |
| Propanolol | 1 (2%) | 0 | 0 | 0 | 0 |
| **3.** Hormone replacement |  |  |  |  |  |
| Levothyroxine sodium | 0 | 1 (9.1%) | 2 (7.1%) | 0 | 3 (5.8%) |
| Lanreotide | 0 | 0 | 1 (3.6%) | 0 | 1 (1.9%) |
| **4**. Antiarrhythmic drugs and cardiac insufficiency |  |  |  |  |  |
| Carvedilol | 1 (2%) | 0 | 1 (3.6%) | 0 | 1 (1.9%) |
| **5.** Neuropathic pain |  |  |  |  |  |
| Gabapentin | 0 | 0 | 0 | 1 (7.7%) | 1 (1.9%) |
| **6.** Renal lithiasis |  |  |  |  |  |
| Allopurinol | 0 | 1 (9.1%) | 2 (7.1%) | 1 (7.7%) | 4 (7.7%) |
| **7**. Antidiabetic agents |  |  |  |  |  |
| Metformin | 0 | 0 | 1 (3.6%) | 0 | 1 (1.9%) |
| Insulin | 1 (2%) | 0 | 0 | 0 | 0 |
| **8.** Antibiotics |  |  |  |  |  |
| Ceftriaxone sodium | 0 | 0 | 1 (3.6%) | 0 | 1 (1.9%) |
| Metronidazole | 0 | 0 | 1 (3.6%) | 0 | 1 (1.9%) |
| Bactrim | 0 | 0 | 1 (3.6%) | 1 (7.7%) | 1 (1.9%) |
| **9.** Supplements |  |  |  |  |  |
| Calcium | 2 (4%) | 0 | 4 (14.3%) | 0 | 4 (7.7%) |
| Potassium Citrate | 1 (2%) | 0 | 1 (3.6%) | 0 | 1 (1.9%) |
| Vitamin D | 3 (6%) | 1 (9.1%) | 1 (3.6%) | 1 (7.7%) | 3 (5.8%) |

Table S3. All the 158 VOMs identified in cerumen samples, their respective match probabilities according to NIST11s library, absolute retention time (Abs), relative retention time (Rel. Rtª) to Internal Standard (IS), CAS No, and the frequency of occurrence in cancer and control group. The cancer samples were divided according to the histological cancer type (Carcinoma, Lymphoma, and Leukemia). The frequency of occurrence was calculated by the presence of each metabolite in each sample. Total cancer group corresponds to the frequency accumulative of the metabolites in each cancer type. The last column (Change) is the difference between Control and Total Cancer Group.

| **Nº** | Volatile Organic Metabolites | Match % | Abs. Rt | Rel. Rt ^a^ | CAS No. | Frequency of occurrence (%) ^b^ | | | | | Change ^d^  (%) |
| --- | --- | --- | --- | --- | --- | --- | --- | --- | --- | --- | --- |
|  |  |  |  |  |  | Lymphoma | Carcinoma | Leukemia | Total Cancer Group^c^ | Control |  |
| **1** | Acetone | 90 | 1.476 | 0.056 | 67 - 64 - 1 | 100.0 | 100.0 | 100.0 | 100.0 | 100.0 | 0.0 |
| **2** | 2-Methylpropanal | 98 | 2.014 | 0.076 | 78 - 84 - 2 | 100.0 | 100.0 | 100.0 | 100.0 | 100.0 | 0.0 |
| **3** | 2.5-Dihydrofuran | 85 | 2.183 | 0.083 | 1708 - 29 - 8 | 9.1 | 3.6 | 23.1 | 9.6 | 74.0 | -64.4 |
| **4** | Butanal | 93 | 2.462 | 0.093 | 123 - 72 - 8 | 90.9 | 82.1 | 100.0 | 88.5 | 84.0 | 4.5 |
| **5** | 2-Butanone | 87 | 2.545 | 0.097 | 78 - 93 - 3 | 90.9 | 96.4 | 100.0 | 96.2 | 58.0 | 38.2 |
| **6** | 2-Methyl-3-buten-2-ol | 83 | 2.829 | 0.107 | 115 - 18 - 4 | 72.7 | 7.1 | 46.2 | 30.8 | 36.0 | -5.2 |
| **7** | Acetic acid | 96 | 3.140 | 0.119 | 64 - 19 - 7 | 27.3 | 75.0 | 46.2 | 57.7 | 32.0 | 25.7 |
| **8** | 3-Methylbutanal | 89 | 3.800 | 0.144 | 590 - 86 - 3 | 100.0 | 100.0 | 92.3 | 98.1 | 98.0 | 0.1 |
| **9** | 2-Methylbutanal | 97 | 4.089 | 0.155 | 96 - 17 - 3 | 100.0 | 100.0 | 100.0 | 100.0 | 96.0 | 4.0 |
| **10** | 3-Methylhexane | 83 | 4.519 | 0.172 | 589 - 34 - 4 | 54.5 | 21.4 | 53.8 | 36.5 | 8.0 | 28.5 |
| **11** | 2-Pentanone | 86 | 4.948 | 0.188 | 107 - 87 - 9 | 54.5 | 92.9 | 46.2 | 73.1 | 86.0 | -12.9 |
| **12** | Pentanal | 87 | 5.299 | 0.201 | 110 - 62 - 3 | 81.8 | 67.9 | 100.0 | 78.9 | 74.0 | 4.9 |
| **13** | Heptane | 97 | 5.487 | 0.208 | 142 - 82 - 5 | 9.1 | 42.9 | 69.2 | 42.3 | 22.0 | 20.3 |
| **14** | 2.5-Dimethyl-2.3-dihydrofuran | 96 | 5.803 | 0.220 | 17108 - 52 - 0 | 54.5 | 39.3 | 30.8 | 40.4 | 36.0 | 4.4 |
| **15** | [4-methylcyclopentane-1.3-dione](http://www.molbase.com/en/cas-35029-03-9.html) | 93 | 6.998 | 0.266 | 35029 - 03 - 9 | 0.0 | 7.1 | 7.7 | 5.8 | 2.0 | 3.8 |
| **16** | Dimethyl disulfide | 86 | 7.510 | 0.285 | 624 - 92 - 0 | 0.0 | 14.3 | 7.7 | 9.6 | 12.0 | -2.4 |
| **17** | 1H-Pyrrole | 93 | 8.594 | 0.326 | 109 - 97 - 7 | 0.0 | 10.7 | 7.7 | 7.7 | 8.0 | -0.3 |
| **18** | Toluene | 95 | 8.868 | 0.337 | 108 - 88 - 3 | 9.1 | 7.1 | 7.7 | 7.7 | 4.0 | 3.7 |
| **19** | 6-Methyl-3.4-dihydro-2H-pyran | 88 | 9.354 | 0.355 | 16015 - 11 - 5 | 0.0 | 3.6 | 0.0 | 1.9 | 4.0 | -2.1 |
| **20** | 2-Methylpropanoic acid | 95 | 10.202 | 0.387 | 79 - 31 - 2 | 18.2 | 3.6 | 7.7 | 7.7 | 4.0 | 3.7 |
| **21** | 2-Hexanone | 91 | 10.488 | 0.398 | 591 - 78 - 6 | 100.0 | 100.0 | 92.3 | 98.1 | 98.0 | 0.1 |
| **22** | Hexanal | 89 | 11.151 | 0.423 | 66 - 25 - 1 | 81.8 | 53.6 | 92.3 | 69.2 | 64.0 | 5.2 |
| **23** | Octane | 81 | 11.322 | 0.430 | 111 - 65 - 9 | 18.2 | 3.6 | 23.1 | 11.5 | 12.0 | -0.5 |
| **24** | 1.5-Dimethyl-6-oxabicyclo[3.1.0]hexane | 91 | 12.333 | 0.468 | 82461 - 31 - 2 | 54.5 | 35.7 | 38.5 | 40.4 | 36.0 | 4.4 |
| **25** | 2-Methylbutanoic acid | 88 | 18.086 | 0.687 | 116 - 53 - 0 | 0.0 | 3.6 | 7.7 | 3.9 | 2.0 | 1.9 |
| **26** | 2-Heptanone | 90 | 19.475 | 0.740 | 110 - 43 - 0 | 90.9 | 85.7 | 92.3 | 88.5 | 84.0 | 4.5 |
| **27** | 2-Ethylbutanal | 92 | 20.350 | 0.772 | 07 - 96 - 1 | 45.5 | 10.7 | 23.1 | 21.2 | 10.0 | 11.2 |
| **28** | 3.4-Dimethyl-2-hexanone | 80 | 20.419 | 0.775 | 19550 - 10 - 8 | 9.1 | 0.0 | 15.4 | 5.8 | 10.0 | -4.2 |
| **29** | Heptanal | 87 | 20.616 | 0.783 | 111 - 71 - 7 | 18.2 | 0.0 | 30.8 | 11.5 | 8.0 | 3.5 |
| **30** | Nonane | 91 | 20.813 | 0.790 | 111 - 84 - 2 | 0.0 | 0.0 | 23.1 | 5.8 | 12.0 | -6.2 |
| **31** | 1-(3-Ethylcyclobutyl)ethanone | 89 | 21.459 | 0.815 | 56335 - 71 - 8 | 9.1 | 10.7 | 15.4 | 11.5 | 4.0 | 7.5 |
| **32** | 6-Methyl-2-heptanone | 90 | 27.943 | 1.061 | 928 - 68 - 7 | 0.0 | 0.0 | 0.0 | NF^e^ | 16.0 | -16.0 |
| **33** | 6-Methyl-5-hepten-2-one | 81 | 31.980 | 1.214 | 110 - 93 - 0 | 45.5 | 7.1 | 15.4 | 17.3 | 10.0 | 7.3 |
| **34** | 2-Methyl-2-cyclohexen-1-one | 81 | 32.303 | 1.227 | 1121 - 18 -2 | 36.4 | 7.1 | 30.8 | 19.2 | 14.0 | 5.2 |
| **35** | 2-Pentylfuran | 93 | 32.520 | 1.235 | 3777 - 69 - 3 | 100.0 | 92.9 | 92.3 | 94.2 | 90.0 | 4.2 |
| **36** | 1-(2.2-Dimethylcyclopentyl)ethanone | 83 | 33.732 | 1.281 | 3664 - 75 - 3 | 54.5 | 21.4 | 53.8 | 36.5 | 26.0 | 10.5 |
| **37** | 2-Methyloctahydropentalene | 80 | 36.100 | 1.371 | 3868 - 64 - 2 | 72.7 | 60.7 | 61.5 | 63.5 | 44.0 | 19.5 |
| **38** | 3-Hydroxy-4.4-dimethyldihydro-2(3H)-furanone | 85 | 37.109 | 1.409 | 599 - 04 - 2 | 18.2 | 10.7 | 7.7 | 11.5 | 4.0 | 7.5 |
| **39** | [2-methyl-N-(2-methylbutyl)butan-1-imine](http://www.molbase.com/en/cas-54518-97-7.html) | 84 | 37.775 | 1.435 | 54518 - 97 - 7 | 9.1 | 14.3 | 7.7 | 11.5 | 6.0 | 5.5 |
| **40** | 1-Methylcyclooctene | 86 | 38.033 | 1.444 | 933 - 11 - 9 | 27.3 | 32.1 | 30.8 | 30.8 | 32.0 | -1.2 |
| **41** | 2-Methyl-2-heptenal | 84 | 38.436 | 1.460 | 30567 - 26 - 1 | 18.2 | 46.4 | 7.7 | 30.8 | 36.0 | -5.2 |
| **42** | 6-Methyl-7-oxabicyclo[4.1.0]heptan-2-one | 83 | 38.453 | 1.460 | 21889 - 89 - 4 | 72.7 | 25.0 | 61.5 | 44.2 | 84.0 | -39.8 |
| **43** | 5-Ethyldihydro-2(3H)-furanone | 87 | 38.585 | 1.465 | 695 - 06 - 7 | 63.6 | 60.7 | 69.2 | 63.5 | 10.0 | 53.5 |
| **44** | 3-Methyl-2-cyclohexen-1-one | 94 | 38.695 | 1.470 | 1193 - 18 - 6 | 0.0 | 32.1 | 15.4 | 21.2 | 6.0 | 15.2 |
| **45** | 1-(2-Methyl-1-cyclopenten-1-yl)ethanone | 93 | 40.455 | 1.536 | 3168 - 90 -9 | 0.0 | 3.6 | 15.4 | 5.8 | NF | 5.8 |
| **46** | 5-Butyl-4-methyldihydro-2(3H)-furanone | 96 | 42.129 | 1.600 | 39638 - 67 - 0 | 18.2 | 21.4 | 23.1 | 21.2 | 18.0 | 3.2 |
| **47** | 6-Methyltetrahydro-2H-pyran-2-one | 96 | 42.403 | 1.610 | 823 - 22 - 3 | 100.0 | 100.0 | 92.3 | 98.1 | 72.0 | 26.1 |
| **48** | 4-Methyltetrahydro-2H-pyran-2-one | 96 | 43.020 | 1.634 | 1121 - 84 - 2 | 81.8 | 89.3 | 61.5 | 80.8 | 58.0 | 22.8 |
| **49** | 2-Nonanone | 96 | 43.046 | 1.635 | 821 - 55- 6 | 0.0 | 3.6 | 23.1 | 7.7 | 4.0 | 3.7 |
| **50** | 2-Oxepanone | 92 | 43.716 | 1.660 | 502 - 44 - 3 | 54.5 | 78.6 | 30.8 | 61.5 | 34.0 | 27.5 |
| **51** | 2-Butyl-1-octanol | 93 | 44.038 | 1.672 | 3913 - 02 - 8 | 0.0 | 3.6 | 0.0 | 1.9 | NF | 1.9 |
| **52** | Nonanal | 86 | 44.091 | 1.674 | 124 - 19 - 6 | 36.4 | 0.0 | 38.5 | 17.3 | 22.0 | -4.7 |
| **53** | 4-tert-Butyl-1-cyclohexene | 95 | 44.332 | 1.684 | 2228 - 98 - 0 | 0.0 | 10.7 | 7.7 | 7.7 | 6.0 | 1.7 |
| **54** | 3-Methyl-3-vinylcyclohexanone | 94 | 44.762 | 1.700 | 68269 - 56 - 7 | 45.5 | 39.3 | 46.2 | 42.3 | 44.0 | -1.7 |
| **55** | 2.5-Pyrrolidinedione | 88 | 46.316 | 1.759 | 123 - 56 - 8 | 63.6 | 35.7 | 38.5 | 42.3 | 14.0 | 28.3 |
| **56** | 5-Propyldihydro-2(3H)-furanone | 95 | 47.926 | 1.820 | 105 - 21 - 5 | 36.4 | 78.6 | 46.2 | 61.5 | 58.0 | 3.5 |
| **57** | 2-Decanone | 91 | 48.862 | 1.856 | 693 - 54 - 9 | 0.0 | 0.0 | 0.0 | NF | 6.0 | -6.0 |
| **58** | 2.5-Dimethylaniline | 87 | 49.092 | 1.864 | 95 - 78 - 3 | 0.0 | 0.0 | 0.0 | NF | 8.0 | -8.0 |
| **59** | 1-Decanol | 96 | 50.301 | 1.910 | 112 - 30 - 1 | 0.0 | 0.0 | 0.0 | NF | 10.0 | -10.0 |
| **60** | Decanal | 89 | 50.608 | 1.922 | 112 - 31- 2 | 36.4 | 10.7 | 23.1 | 19.2 | 12.0 | 7.2 |
| **61** | 6-Ethyltetrahydro-2H-pyran-2-one | 97 | 50.802 | 1.929 | 3301 - 90 - 4 | 90.9 | 89.3 | 92.3 | 90.4 | 64.0 | 26.4 |
| **62** | [1-(3.4-dihydro-2H-pyridin-1-yl)ethanone](http://www.molbase.com/en/cas-19615-27-1.html) | 83 | 51.072 | 1.940 | 19615 - 27 - 1 | 0.0 | 0.0 | 0.0 | NF | 8.0 | -8.0 |
| **63** | 1-Methyl-3-(2-methyl-1-propenyl)cyclopentane | 86 | 51.249 | 1.946 | 75873 - 01 - 7 | 100.0 | 100.0 | 92.3 | 98.1 | 90.0 | 8.1 |
| **64** | 2-Undecanone | 87 | 51.779 | 1.966 | 112 - 12 - 9 | 45.5 | 28.6 | 46.2 | 36.5 | 28.0 | 8.5 |
| **65** | Dodecane | 97 | 52.617 | 1.998 | 112 - 40 - 3 | 18.2 | 14.3 | 23.1 | 17.3 | 20.0 | -2.7 |
| **66** | 1-Pentyl-1H-pyrrole | 96 | 53.016 | 2.013 | 699 - 22 - 9 | 36.4 | 10.7 | 38.5 | 23.1 | 28.0 | -4.9 |
| **67** | (Isopropylsulfanyl)benzene | 81 | 53.625 | 2.036 | 3019 - 20 - 3 | 0.0 | 25.0 | 23.1 | 19.2 | 4.0 | 15.2 |
| **68** | 1-(2-Methyl-1-propen-1-yl)pyrrolidine | 83 | 55.623 | 2.112 | 2403 - 57 - 8 | 9.1 | 10.7 | 15.4 | 11.5 | 24.0 | -12.5 |
| **69** | 5-Butyldihydro-2(3H)-furanone | 82 | 56.506 | 2.146 | 104 - 50 - 7 | 27.3 | 50.0 | 46.2 | 44.2 | 4.0 | 40.2 |
| **70** | 6.7.8.9-Tetrahydro-5H-cyclohepta[b]pyridine | 84 | 58.291 | 2.214 | 7197 - 96 - 8 | 0.0 | 3.6 | 7.7 | 3.9 | 2.0 | 1.9 |
| **71** | Nonanoic acid | 86 | 58.500 | 2.222 | 112 - 05 - 0 | 0.0 | 14.3 | 30.8 | 15.4 | 12.0 | 3.4 |
| **72** | 6-Propyltetrahydro-2H-pyran-2-one | 83 | 58.548 | 2.223 | 698 - 76 - 0 | 54.5 | 89.3 | 38.5 | 69.2 | 24.0 | 45.2 |
| **73** | 1H-Indole | 80 | 59.440 | 2.257 | 120 - 72 - 9 | 36.4 | 32.1 | 69.2 | 42.3 | 38.0 | 4.3 |
| **74** | 6.10-Dimethyl-2-undecanone | 93 | 59.610 | 2.264 | 1604 - 34 - 8 | 45.5 | 25.0 | 46.2 | 34.6 | 44.0 | -9.4 |
| **75** | Undecanal | 96 | 60.580 | 2.301 | 112 - 44 - 7 | 9.1 | 3.6 | 30.8 | 11.5 | 22.0 | -10.5 |
| **76** | Tridecane | 96 | 60.601 | 2.301 | 629 - 50 - 5 | 63.6 | 50.0 | 38.5 | 50.0 | 56.0 | -6.0 |
| **77** | 2-(2-Methylpropylidene)cycloheptanone | 96 | 60.815 | 2.310 | 120694 - 89 - 5 | 0.0 | 32.1 | 15.4 | 21.2 | 8.0 | 13.2 |
| **78** | (4α.4aα.8aβ)Octahydro-4.8a-dimethyl-4a(2H)-naphthalenol | 96 | 62.874 | 2.388 | 19700 - 21 - 1 | 9.1 | 14.3 | 38.5 | 19.2 | 18.0 | 1.2 |
| **79** | 2-Isopropyl-5-methylcyclohexanone | 92 | 63.271 | 2.403 | 1074-95-9 | 54.5 | 35.7 | 53.8 | 44.2 | 36.0 | 8.2 |
| **80** | 5-Pentyldihydro-2(3H)-furanone | 93 | 63.422 | 2.409 | 104 - 61 - 0 | 100.0 | 71.4 | 76.9 | 78.9 | 48.0 | 30.9 |
| **81** | 4-Isopropyl-2.6-dimethyl-4-heptanol | 80 | 63.867 | 2.425 | 54775 - 01 - 8 | 63.6 | 42.9 | 53.8 | 50.0 | 44.0 | 6.0 |
| **82** | 2.6-Dimethyl-4-propyl-4-heptanol | 81 | 63.878 | 2.426 | 54774 - 83 - 3 | 9.1 | 71.4 | 46.2 | 51.9 | 48.0 | 3.9 |
| **83** | Undecanoic acid | 90 | 64.014 | 2.431 | 112 - 37 - 8 | 63.6 | 64.3 | 46.2 | 59.6 | 28.0 | 31.6 |
| **84** | Dioctyl ether | 96 | 64.370 | 2.445 | 629 - 82 - 3 | 45.5 | 25.0 | 46.2 | 34.6 | 34.0 | 0.6 |
| **85** | 6-Butyltetrahydro-2H-pyran-2-one | 92 | 64.719 | 2.458 | 3301 - 94 - 8 | 81.8 | 75.0 | 84.6 | 78.9 | 38.0 | 40.9 |
| **86** | 3.4-Dihydro-1(2H)-quinolinecarbaldehyde | 92 | 65.160 | 2.475 | 2739 - 16 - 4 | 45.5 | 21.4 | 46.2 | 32.7 | 42.0 | -9.3 |
| **87** | Tetradecane | 92 | 65.304 | 2.480 | 629 - 59 - 4 | 81.8 | 85.7 | 92.3 | 86.5 | 68.0 | 18.5 |
| **88** | 3-Phenylthiophene | 87 | 65.556 | 2.490 | 2404 - 87 - 7 | 72.7 | 14.3 | 53.8 | 36.5 | 34.0 | 2.5 |
| **89** | 1.3-dihydro-3.3-dimethyl-2H-Indol-2-one | 91 | 66.104 | 2.510 | 19155 - 24 - 9 | 9.1 | 10.7 | 30.8 | 15.4 | 8.0 | 7.4 |
| **90** | 1.4a-Dimethyloctahydro-2(1H)-naphthalenone | 89 | 66.256 | 2.516 | 22738 - 31 - 4 | 81.8 | 85.7 | 92.3 | 86.5 | 78.0 | 8.5 |
| **91** | 6.10-Dimethyl-undeca-5.9-dien-2-one | 90 | 67.026 | 2.545 | 689 - 67 - 8 | 81.8 | 82.1 | 84.6 | 82.7 | 72.0 | 10.7 |
| **92** | 5-Methyl-5-hexen-2-one | 94 | 67.291 | 2.555 | 3240 - 09 - 3 | 18.2 | 7.1 | 15.4 | 11.5 | 18.0 | -6.5 |
| **93** | 5-Hexyldihydro-2(3H)-furanone | 88 | 67.470 | 2.562 | 706 - 14 - 9 | 63.6 | 46.4 | 76.9 | 57.7 | 46.0 | 11.7 |
| **94** | 1-Tridecene | 95 | 67.862 | 2.577 | 112 - 53 - 8 | 36.4 | 46.4 | 69.2 | 50.0 | 42.0 | 8.0 |
| **95** | 4a-Methyldecahydro-2H-benzo[7]annulen-2-one | 91 | 67.935 | 2.580 | 55103 - 64 - 5 | 63.6 | 46.4 | 23.1 | 44.2 | 42.0 | 2.2 |
| **96** | 6-Pentyltetrahydro-2H-pyran-2-one | 89 | 68.506 | 2.602 | 705 - 86 - 2 | 100.0 | 82.1 | 92.3 | 88.5 | 72.0 | 16.5 |
| **97** | Hexadecane | 81 | 68.598 | 2.605 | 544 - 76 - 3 | 27.3 | 42.9 | 30.8 | 36.5 | 12.0 | 24.5 |
| **98** | 4-Butylpyridine | 91 | 68.656 | 2.607 | 5335 - 75 - 1 | 90.9 | 92.9 | 76.9 | 88.5 | 66.0 | 22.5 |
| **99** | N-(3-Acetylphenyl)acetamide | 88 | 68.764 | 2.611 | 7463 - 31 - 2 | 18.2 | 25.0 | 23.1 | 23.1 | 54.0 | -30.9 |
| **100** | Tetrahydro-2H-pyran-2-yl-methanol | 90 | 68.918 | 2.617 | 100 - 72 - 1 | 27.3 | 28.6 | 46.2 | 32.7 | 30.0 | 2.7 |
| **101** | 5.5-Dimethyl-2.4-hexanedione | 92 | 69.048 | 2.622 | 104 - 61 - 0 | 45.5 | 42.9 | 23.1 | 38.5 | 32.0 | 6.5 |
| **102** | 2.3-Dimethylquinoline | 88 | 69.124 | 2.625 | 1721 - 89 - 7 | 0.0 | 17.9 | 15.4 | 13.5 | 34.0 | -20.5 |
| **103** | 1-Dodecanol | 87 | 69.411 | 2.636 | 112 - 53 - 8 | 18.2 | 7.1 | 30.8 | 15.4 | 16.0 | -0.6 |
| **104** | 1-Cyclododecylethanone | 91 | 69.796 | 2.651 | 28925 - 00 - 0 | 27.3 | 3.6 | 38.5 | 17.3 | 30.0 | -12.7 |
| **105** | Dodecanoic acid | 89 | 70.257 | 2.668 | 143 - 07 - 7 | 54.5 | 60.7 | 53.8 | 57.7 | 22.0 | 35.7 |
| **106** | Tridecanoic Acid | 90 | 70.406 | 2.674 | 629 - 66 - 3 | 18.2 | 0.0 | 30.8 | 11.5 | 26.0 | -14.5 |
| **107** | 1-Pentyl-4-(4-propylcyclohexyl)-1-cyclohexene | 90 | 70.839 | 2.690 | 62337 - 96 - 6 | 18.2 | 25.0 | 46.2 | 28.9 | 26.0 | 2.9 |
| **108** | 2.3.7-Trimethyl-1H-indole | 98 | 71.087 | 2.700 | 27505 - 78 - 8 | 63.6 | 17.9 | 53.8 | 36.5 | 30.0 | 6.5 |
| **109** | 2-Tetradecanone | 85 | 71.132 | 2.701 | 2345 - 27 - 9 | 72.7 | 53.6 | 84.6 | 65.4 | 72.0 | -6.6 |
| **110** | Octadecane | 93 | 71.228 | 2.705 | 593 - 45 - 3 | 100.0 | 78.6 | 69.2 | 80.8 | 92.0 | -11.2 |
| **111** | 7-Decen-2-one | 87 | 71.372 | 2.710 | 35194 - 33 - 3 | 0.0 | 0.0 | 0.0 | NF | 6.0 | -6.0 |
| **112** | 1.1-Cyclohexanedimethanol | 96 | 71.492 | 2.715 | 2658 - 60 - 8 | 81.8 | 75.0 | 84.6 | 78.9 | 76.0 | 2.9 |
| **113** | (3-Methyl-2-cyclohexen-1-yl)acetaldehyde | 89 | 71.591 | 2.719 | 129993 - 40 - 4 | 100.0 | 96.4 | 92.3 | 96.2 | 94.0 | 2.2 |
| **114** | N.N-Dibutylaniline | 97 | 71.767 | 2.725 | 613- 29 - 6 | 9.1 | 14.3 | 15.4 | 13.5 | 30.0 | -16.5 |
| **115** | 6.6.8a-Trimethyloctahydroindeno[1.7a-b]oxete | 83 | 71.963 | 2.733 | 43125 - 92 - 4 | 100.0 | 100.0 | 100.0 | 100.0 | 100.0 | 0.0 |
| **116** | (1Z)-2-Ethyl-N-hydroxycyclohexanimine | 86 | 72.078 | 2.737 | 86823 - 11 - 2 | 100.0 | 92.9 | 84.6 | 92.3 | 88.0 | 4.3 |
| **117** | 3.5-Diisopropenyl-1.1.2-trimethylcyclohexane | 87 | 72.181 | 2.741 | 62337 - 96 - 6 | 27.3 | 60.7 | 46.2 | 50.0 | 36.0 | 14.0 |
| **118** | 1.1.4.7-Tetramethyldecahydro-1H-cycloprop[e]azulene | 97 | 72.328 | 2.747 | 6790 - 78 - 9 | 27.3 | 46.4 | 53.8 | 44.2 | 50.0 | -5.8 |
| **119** | 2.6.10-Trimethyldodecane | 84 | 72.394 | 2.749 | 3891 - 98 - 3 | 27.3 | 64.3 | 46.2 | 51.9 | 36.0 | 15.9 |
| **120** | 1-(Decyloxy)decane | 86 | 72.603 | 2.757 | 2456 - 28 - 2 | 45.5 | 42.9 | 30.8 | 40.4 | 50.0 | -9.6 |
| **121** | 2-Methyl-1-hexadecanol | 82 | 72.711 | 2.761 | 2490 - 48 - 4 | 81.8 | 92.9 | 61.5 | 82.7 | 78.0 | 4.7 |
| **122** | 2.6.10.15-Tetramethylheptadecane | 83 | 72.854 | 2.767 | 54833 - 48 - 6 | 36.4 | 28.6 | 38.5 | 32.7 | 38.0 | -5.3 |
| **123** | (2E)-2.6-Dimethyl-2.7-ocadiene-1.6-diol | 90 | 73.016 | 2.773 | 64142 - 78 - 5 | 36.4 | 17.9 | 61.5 | 32.7 | 18.0 | 14.7 |
| **124** | 2,6-Dimethyloctadecane | 93 | 73.321 | 2.784 | 75163 - 97 - 2 | 27.3 | 25.0 | 15.4 | 23.1 | 16.0 | 7.1 |
| **125** | 2 -Pentadecanone | 96 | 73.475 | 2.790 | 2345 - 28 - 0 | 18.2 | 64.3 | 53.8 | 51.9 | 28.0 | 23.9 |
| **126** | Eicosane | 96 | 73.508 | 2.792 | 112 - 95 - 8 | 81.8 | 39.3 | 92.3 | 61.5 | 96.0 | -34.5 |
| **127** | 2-Octyl-1-decanol | 96 | 73.659 | 2.797 | 45235-48-1 | 54.5 | 85.7 | 69.2 | 75.0 | 58.0 | 17.0 |
| **128** | 6-Heptyltetrahydro-2H-pyran-2-one | 96 | 73.806 | 2.803 | 713 - 95 - 1 | 100.0 | 67.9 | 69.2 | 75.0 | 98.0 | -23.0 |
| **129** | 2-Methyl-4-(2.6.6-trimethyl-1-cyclohexen-1yl)-1-butenylformate | 92 | 73.994 | 2.810 | 21730 - 91 - 6 | 18.2 | 42.9 | 23.1 | 32.7 | 40.0 | -7.3 |
| **130** | n-Tetradecanoic acid | 93 | 74.000 | 2.810 | 544 - 63 - 8 | 36.4 | 17.9 | 46.2 | 28.9 | NF | 28.9 |
| **131** | 1-Octadecanol | 87 | 74.248 | 2.820 | 112 - 92 - 5 | 9.1 | 0.0 | 23.1 | 7.7 | 18.0 | -10.3 |
| **132** | 2-Hydroxycyclononanone | 96 | 74.470 | 2.828 | 496 - 83 - 3 | 36.4 | 3.6 | 53.8 | 23.1 | 28.0 | -4.9 |
| **133** | 2.5-Dipropyltetrahydrofuran | 89 | 74.532 | 2.830 | 4457 - 62 - 9 | 81.8 | 75.0 | 61.5 | 73.1 | 58.0 | 15.1 |
| **134** | 7-Octadecanone | 97 | 74.651 | 2.835 | 18277 - 00 - 4 | 36.4 | 28.6 | 23.1 | 28.9 | 34.0 | -5.2 |
| **135** | n-Pentadecanoic acid | 83 | 74.872 | 2.843 | 1002 - 84 - 2 | 100.0 | 85.7 | 69.2 | 84.6 | 80.0 | 4.6 |
| **136** | n-Hexadecanoic acid | 86 | 75.020 | 2.849 | 57 - 10 - 3 | 36.4 | 21.4 | 38.5 | 28.9 | 34.0 | -5.2 |
| **137** | 2-Nonadecanone | 87 | 75.981 | 2.886 | 629 - 66 - 3 | 100.0 | 78.6 | 92.3 | 86.5 | 88.0 | -1.5 |
| **138** | 2-Ethylhexyl salicylate | 90 | 76.398 | 2.901 | 118 - 60 - 5 | 18.2 | 10.7 | 23.1 | 15.4 | 10.0 | 5.4 |
| **139** | 2-Butyltetrahydrofuran | 91 | 76.588 | 2.909 | 1004 - 29 - 1 | 0.0 | 10.7 | 30.8 | 13.5 | 14.0 | -0.5 |
| **140** | Isopropyl myristate | 90 | 76.647 | 2.911 | 110 - 27 - 0 | 9.1 | 14.3 | 23.1 | 15.4 | 12.0 | 3.4 |
| **141** | n-Octadecanoic acid | 92 | 76.832 | 2.918 | 57- 11- 4 | 18.2 | 10.7 | 30.8 | 17.3 | 34.0 | -16.7 |
| **142** | n-Octadecanal | 88 | 77.219 | 2.933 | 638- 66 -4 | 9.1 | 3.6 | 15.4 | 7.7 | 16.0 | -8.3 |
| **143** | 2-Methyl-7-nonadecene | 80 | 77.337 | 2.937 | 219750 - 68 -2 | 45.5 | 3.6 | 46.2 | 23.1 | 34.0 | -10.9 |
| **144** | 2-Hydroxycyclopentadecanone | 90 | 77.554 | 2.945 | 4727 - 18 - 8 | 18.2 | 25.0 | 61.5 | 32.7 | 62.0 | -29.3 |
| **145** | n-Nonadecanoic acid | 93 | 77.717 | 2.951 | 646 - 30 - 0 | 63.6 | 75.0 | 46.2 | 65.4 | 12.0 | 53.4 |
| **146** | 1 -tert-Butoxy-1-cycloheptene | 92 | 77.918 | 2.959 | 49565 - 07 - 3 | 9.1 | 25.0 | 46.2 | 26.9 | 12.0 | 14.9 |
| **147** | Diisobutyl phthalate | 88 | 78.122 | 2.967 | 84 - 69 - 5 | 54.5 | 78.6 | 84.6 | 75.0 | 34.0 | 41.0 |
| **148** | 4-Cyclohexyl-2-butanone | 89 | 78.388 | 2.977 | 2316 - 85 - 0 | 63.6 | 17.9 | 61.5 | 38.5 | 34.0 | 4.5 |
| **149** | 6.10.14-Trimethyl-2-pentadecanone | 91 | 79.207 | 3.008 | 502 - 69 - 2 | 90.9 | 32.1 | 100.0 | 61.5 | 88.0 | -26.5 |
| **150** | Methyl palmitate | 92 | 79.942 | 3.036 | 112 - 39 - 0 | 63.6 | 64.3 | 53.8 | 61.5 | 38.0 | 23.5 |
| **151** | cis-10-Nonadecenoic acid | 93 | 80.380 | 3.053 | 73033 - 09 -7 | 63.6 | 75.0 | 76.9 | 73.1 | 72.0 | 1.1 |
| **152** | Eicosanoic acid | 94 | 80.983 | 3.075 | 506 - 30 - 9 | 100.0 | 100.0 | 100.0 | 100.0 | 100.0 | 0.0 |
| **153** | 1-(Vinyloxy)hexadecane | 88 | 84.839 | 3.222 | 822 - 28 - 6 | 54.5 | 32.1 | 61.5 | 44.2 | 30.0 | 14.2 |
| **154** | 1-(Vinyloxy)octadecane | 84 | 84.973 | 3.227 | 930 - 02 - 9 | 27.3 | 21.4 | 30.8 | 25.0 | 58.0 | -33.0 |
| **155** | Butyl palmitate | 96 | 86.840 | 3.298 | 111 - 06 - 8 | 100.0 | 100.0 | 100.0 | 100.0 | 98.0 | 2.0 |
| **156** | Butyl stearate | 94 | 91.480 | 3.474 | 123 - 95 - 5 | 100.0 | 100.0 | 100.0 | 100.0 | 96.0 | 4.0 |
| **157** | Bis(2-ethylhexyl) phthalate | 95 | 96.817 | 3.677 | 117 - 81 - 7 | 90.9 | 89.3 | 84.6 | 88.5 | 46.0 | 42.5 |
| **158** | Squalene | 90 | 97.176 | 3.690 | 111 - 02 - 4 | 100.0 | 96.4 | 100.0 | 98.1 | 84.0 | 14.1 |

^a^ Relative to the retention time of the 3-methylcyclohexanone (IS). ^b^ Calculated by the count of the presence of the metabolite in the samples of each group (Lymphoma n = 11, Carcinoma n = 28, Leukemia n = 13, Total Cancer Group n = 52, and Control Group n = 50). ^c^ Relative to the count presence in all 52 cancer samples. ^d^ Percentage of change between the Total Cancer and Control Groups. ^e^ NF = Not Found, means that the metabolites were not detected in any sample for cancer or control group.

**Table S4.** Chemical Structures of the 27 VOMs selected by GA and PLS as potential biomarkers for cancer in cerumen. The VOMs are separated by superclasses (organooxygen, carboxylic acids, organosulfur, organonitrogen, and hydrocarbons) and their respective organic class.

| **Organooxygen** | | | | |
| --- | --- | --- | --- | --- |
| Ketones | | | | |
|   **VOM 5** |   **VOM** **134** | |   **VOM** **11** | |
|  |  |  |  |  |
|  |  |  |  |  |
|  |  |  |  |  |
|  |  |  |  |  |
|  |  |  |  |  |
|  |  |  |  |  |
| Aldehydes | Alcohols and derivatives | | | |
| **VOM** **142** |   **VOM 6** |   **VOM** **103** | | **VOM 59** |
|  |  |  |  |  |
|  |  |  |  |  |
|  |  |  |  |  |
|  |  |  |  |  |
|  |  |  |  |  |
|  |  |  |  |  |
|  |  |  |  |  |
| Furanic, lactones and derivatives compounds | | | Epoxides/Oxabicyclo derivatives | |
|   **VOM 3** |   **VOM 43** | | **VOM 42** | |
|  |  |  |  |  |
|  |  |  |  |  |
|  |  |  |  |  |
|  |  |  |  |  |
|  |  |  |  |  |
|  |  |  |  |  |
| Pyran Compounds | | | | |
| **VOM 47** | **VOM 72** | **VOM 85** |   **VOM 128** | |
|  |  |  |  |  |
|  |  |  |  |  |
|  |  |  |  |  |
|  |  |  |  |  |
|  |  |  |  |  |
|  |  |  |  |  |
| Esters and ethers | | | | |
|   **VOM 147** |   **VOM 120** | |   **VOM 157** | |
|  |  |  |  |  |
|  |  |  |  |  |
|  |  |  |  |  |
|  |  |  |  |  |
|  |  |  |  |  |
|  |  |  |  |  |
| **Organic acids and derivatives** | | | | |
| Carboxylic acids | | | | |
| **VOM** **105** |   **VOM 141** | | **VOM 130** | |
|  |  |  |  |  |
|  |  |  |  |  |
|  |  |  |  |  |
|  |  |  |  |  |
|  |  |  |  |  |
|  |  |  |  |  |
| **Organonitrogen compounds** | | | **Organosulfur compounds** | |
| Amines and amides derivatives | | | Dialkyldisulfides and thioethers | |
|   **VOM 58** |   **VOM 99** |   **VOM 102** |   **VOM 88** | |
|  |  |  |  |  |
|  |  |  |  |  |
|  |  |  |  |  |
|  |  |  |  |  |
|  |  |  |  |  |
|  |  |  |  |  |
| **Hydrocarbons** | | | | |
| Alkanes, alkenes, benzene and substituted derivatives | | | | |
|   **VOM 10** |   **VOM 126** | |   **VOM 40** | |
